# Identifying micro-inversions using high-throughput sequencing reads

**DOI:** 10.1186/s12864-015-2305-7

**Published:** 2016-01-11

**Authors:** Feifei He, Yang Li, Yu-Hang Tang, Jian Ma, Huaiqiu Zhu

**Affiliations:** State Key Laboratory for Turbulence and Complex Systems and Department of Biomedical Engineering, and Center for Quantitative Biology, Peking University, Beijing, 100871 China; Department of Bioengineering, University of Illinois, Urbana, IL 61801 USA; Carl R. Woese Institute for Genomic Biology, University of Illinois, Urbana, IL 61801 USA; Division of Applied Mathematics, Brown University, Providence, RI 02912 USA

**Keywords:** Micro-inversion, Next-generation sequencing, Structural variation

## Abstract

**Background:**

The identification of inversions of DNA segments shorter than read length (e.g., 100 bp), defined as micro-inversions (MIs), remains challenging for next-generation sequencing reads. It is acknowledged that MIs are important genomic variation and may play roles in causing genetic disease. However, current alignment methods are generally insensitive to detect MIs. Here we develop a novel tool, MID (Micro-Inversion Detector), to identify MIs in human genomes using next-generation sequencing reads.

**Results:**

The algorithm of MID is designed based on a dynamic programming path-finding approach. What makes MID different from other variant detection tools is that MID can handle small MIs and multiple breakpoints within an unmapped read. Moreover, MID improves reliability in low coverage data by integrating multiple samples. Our evaluation demonstrated that MID outperforms Gustaf, which can currently detect inversions from 30 bp to 500 bp.

**Conclusions:**

To our knowledge, MID is the first method that can efficiently and reliably identify MIs from unmapped short next-generation sequencing reads. MID is reliable on low coverage data, which is suitable for large-scale projects such as the 1000 Genomes Project (1KGP). MID identified previously unknown MIs from the 1KGP that overlap with genes and regulatory elements in the human genome. We also identified MIs in cancer cell lines from Cancer Cell Line Encyclopedia (CCLE). Therefore our tool is expected to be useful to improve the study of MIs as a type of genetic variant in the human genome. The source code can be downloaded from: http://cqb.pku.edu.cn/ZhuLab/MID.

**Electronic supplementary material:**

The online version of this article (doi:10.1186/s12864-015-2305-7) contains supplementary material, which is available to authorized users.

## Background

Genomic structural variations (SVs), including insertion, deletion, rearrangement, and duplication are important sources of human genetic diversity and may be responsible for human diseases [1–4]. With the rapid development of high-throughput sequencing technologies, computational tools have been developed to utilize next-generation sequencing data to identify SVs, such as BreakDancer [5], Pindel [6], PRISM [7], DELLY [8], CNVnator [9] and Gustaf [10]. It has been noted that small inversions may provide important insights into genome evolution and disease mechanisms [4, 11]. Previous studies have reported the discovery of small inversions by comparing the human genome with other mammalian genomes [12, 13]. However, despite the progress of computational tools using NGS data to identify SVs, our understanding and strategies for characterizing small inversions remain limited [14]. In this work, we focus on micro-inversions (MIs), defined as small inversions shorter than 100 bp, which are mostly ignored by existing methods.

Computational tools for SV detection mostly rely on read mapping. However, we found that reads with potential MIs, which cannot be mapped to the reference genome initially, are usually discarded as “unmapped reads” by existing alignment tools. As no mapping information is available for the initially unmapped reads, most existing tools are incapable to detect MIs shorter than the read length. Generally speaking, existing methods use features such as read depth, read pair information, and split reads to identify SVs [15]. Yet there are still several challenges to use these features to detect MIs. The small size of MIs makes the identification difficult. The approaches used in methods based on read depth, e.g., CNVnator, would perform poorly with the small size of MIs without locating the breakpoints. The strategies in utilizing read pairs (e.g., BreakDancer, DELLY) and split reads (e.g., Pindel, PRISM, Gustaf) are also not appropriate to identify MIs less than 100 bp, because the size of SVs detected by these tools is dependent on insert size of paired-end reads and insensitive to variants as small as MIs. Additionally, to increase the applicability of the method, it is important to handle datasets with low coverage. For large-scale projects such as the 1000 Genomes Project (1KGP), there is increasing number of low coverage data [16]. For example, the sample NA12878, provided by 1KGP from two paired-end libraries with 15X coverage, has been frequently selected by variant detection tools for performance comparisons (642 deletions, 271 duplications and 30 insertions) [17]. However, most of the human individual genomes were generated with a low coverage of 2–4X in 1KGP so far. Finally, it is also challenging to identify MIs in a read with multiple variants, as other types of variants (e.g., single nucleotide variations (SNVs), small indels and other rearrangements), may also occur in nearby regions of the MI.

In this paper, we focus on MI identification from unmapped NGS short reads. Our algorithm, named MID (Micro-Inversion Detector), specifically detects MIs shorter than the read length. Moreover, MID can be applied to very low coverage samples. What makes MID different from other variant detection tools is that it is sensitive to very small size MIs, and capable of detecting MIs with multiple breakpoints in one read. Moreover, MID proves reliability in low coverage data by integrating multiple samples. The simulation results showed that MID can detect MIs efficiently and reliably using unmapped NGS short reads with low false positives. By applying MID to 1KGP data, we identified 721 MIs, 349 of which are intergenic, 342 MIs are intronic, and 30 MIs are exonic. We also applied MID to the Lung Squamous Cell Carcinoma whole exome sequencing (WXS) data from CCLE, where we identified 12 MIs.

## Results

### Performance of MI detection on simulated data

Until recently, few attempts have been made for MI detection using NGS data. To the best of our knowledge, only a recent tool called Gustaf [10], can detect inversions from 30 bp to 500 bp, which in principle can recognize MIs shorter than the read length. Gustaf is a tool based on a split-read approach, which records the local alignments provided by other tools and draws a split-read graph to use standard graph algorithms to evaluate relationships of the alignments. In addition, it should be noted that no suitable real datasets with confident annotation are available as a benchmark. We therefore used simulated datasets to evaluate the performance of MID. Two different types of simulated datasets were generated in the current work: one with 1,000 MIs only (Dataset 1), and the other one with 1,000 MIs surrounded by other types of variants (Dataset 2). For Dataset 1, we simulated 1,000 MIs ranging from 15 bp to 40 bp, following a normal distribution for MI size, randomly on the whole chr10 (135 Mb) from the human genome assembly hg19 [18]. For Dataset 2, we simulated 1,000 MIs surrounded by 4,000 other structural variants (including SNVs, deletions, insertions, and duplications) following a normal distribution for MI size randomly on chr10, with a size range of 15–40 bp. We also simulated Illumina paired-end short reads (in 76 bp; same as what the sample NA19213 from the 1KGP has) using Maq [19] with an error rate 0.02. Both simulated datasets had 10 different sub-datasets with coverage varying from 2X to 60X (see Table 1). We then ran the Burrows-Wheeler alignment (BWA) tool [20] in the same way as in the 1KGP to get unmapped short reads.

**Table 1:** Sensitivity (SN), Positive Predictive Value (PPV) and Standard Deviation (SD) for MIs simulated onto chr10

	Dataset 1				Dataset 2			
Coverage	MID		Gustaf		MID		Gustaf	
	SN	PPV	SN	PPV	SN	PPV	SN	PPV
2	0.692	0.900	0.423	0.688	0.741	0.976	0.333	0.783
4	0.732	0.911	0.339	0.760	0.840	0.932	0.506	0.872
6	0.782	0.871	0.359	0.718	0.782	0.935	0.427	0.810
8	0.870	0.918	0.403	0.689	0.784	0.916	0.302	0.824
10	0.771	0.857	0.239	0.703	0.877	0.932	0.351	0.800
20	0.767	0.904	0.233	0.667	0.849	0.914	0.267	0.705
30	0.859	0.886	0.188	0.514	0.821	0.912	0.254	0.627
40	0.781	0.860	0.250	0.588	0.820	0.907	0.230	0.603
50	0.781	0.856	0.211	0.654	0.837	0.906	0.266	0.616
60	0.835	0.857	0.264	0.563	0.863	0.900	0.231	0.509
SD	0.052	0.023	0.079	0.072	0.039	0.021	0.086	0.114

To have a clear view of our evaluation results, we define several metrics. If at least one simulated read generated from the reference sequence cover the MI, we call the MI as “detectable MI”. If 80 % of both the detected MI and the original MI overlap, we call the MI as “correctly detected MI” [10]. We then calculate the sensitivity (SN) as the ratio of correctly detected MIs over all detectable MIs and positive predictive value (PPV) as the ratio of correctly detected MIs over all detected MIs.

As mentioned above, unmapped short reads have not been well studied by most existing tools including Gustaf. However, it is very important for the analysis of personalized NGS data to study unmapped short reads so that we can exploit more SV information especially for MIs. Therefore, preprocessing of unmapped reads is needed. After running BWA to get unmapped short reads, we aligned unmapped reads to chr10 the same as what we did in MID, and then recorded the anchored alignment results for Gustaf, including the re-aligned reads and the corresponding reference sequence on chr10. Afterwards the target regions from both re-aligned read and reference were used as the input for Gustaf single-end detecting. For MID, we can directly run the whole pipeline with unmapped short reads discarded by BWA.

Table 1 shows the comparison between MID and Gustaf. Note that the sub-datasets in 2X and 4X coverage are most similar to the real data we got from the 1KGP. The high SN and high PPV of MID in both Dataset 1 and Dataset 2 demonstrates high accuracy of MID in detecting MIs, even with different variants around. Gustaf demonstrates significantly lower SN and PPV in the simulated data. Furthermore, lower standard deviation (SD) of MID compared with Gustaf in simulated data shows stable performance of MID. Overall, MID outperforms Gustaf in identifying MIs.

### Identifying MIs in 1000 genomes project data

The 1KGP provides large number of individual human genomes data with NGS reads [16]. At present, 1KGP data has been widely used for SV detection by existing tools [5, 8, 16]. However, analysis on MIs is still lacking. To detect MIs, MID was applied on population-scale sequencing data from the 1KGP based on publicly released unmapped BAM files [16]. By running MID with reference human genome assembly hg19, MID reports the detailed alignment of each read containing MIs and a list of unique MIs detected. For a typical sample with a total number of 13.5 M unmapped short reads (NA19917), it takes 12 min to run MID with 16 CPU threads.

Generally speaking, low coverage raises problems for MI detection since less support from reads may lead to unreliable results. However, many datasets from large-scale projects (e.g., the 1KGP) are in low coverage. Therefore, it is very useful to develop methods for identifying MIs reliably and efficiently based on multiple low-coverage samples. It has been suggested that integrated analysis using multiple samples can be helpful in improving the reliability of SV detection [11]. We assessed the performance of MID on a total of 770 Illumina samples from the 1KGP [16], which have been categorized by different populations, and then integrated the results at the population level for more reliable and informative results. We eventually focused on 638 samples (full sample list in sample list of Additional file 1), in which MID reported at least one MI.

We calculated the number of unique MIs and the number of reads supporting each MI first. In the following analysis, if not specified, the number of MIs is equal to the number of unique MIs, either in one particular sample or in one population. Moreover, if one MI is supported by multiple reads, we calculate the number of reads containing the same MI as its “occurrence”. Altogether, MID reported 2,413 occurrences of 721 MIs in 638 samples (full list of MIs and annotations refer to Additional file 1: Table S1). Of the 721 detected MIs, 349 are intergenic, 342 MIs are intronic, and 30 MIs are exonic, including five MIs overlapping with CDS regions, seven MIs overlapping with UTR regions, two MIs overlapping with both CDS and UTR regions, and the rest overlapping with miRNA, Mt_rRNA etc. (more details can be found in Additional file 1: Table S1), annotated by GENCODE [21]. Using ENCODE annotations [22], we identified 13 MIs overlapping with proximal transcription factor binding sites (TFBS), and 48 MIs overlapping with distal TFBS following ChIP peaks of transcription factors. As many regulatory elements are in intronic regions [22, 23], plus the effect of MIs extends beyond the inverted regions [24], thus 342 MIs found overlapping with introns, as well as 19 MIs found locate within the 2,000 bp upstream genomic regions of genes can also be informative for further analysis.

Furthermore, the individual samples used by MID are categorized by populations, and populations are grouped by the predominant component of ancestry as follows: East Asia (CDX, CHB, CHS, JPT, and KHV), South Asia (GIH), Europe (CEU, FIN, GBR, IBS, and TSI), America (CLM, MXL, PEL, and PUR), and Africa (YRI, LWK, ASW, and ACB) [16]. Overall MID reported 206 MIs in Americas Group, 262 MIs in East Asia Group, 22 MIs in South Asia Group, 223 MIs in Europe Group, and 284 MIs in Africa Group. Table 2 presents the overview of MIs detected in 638 individual samples, illustrating an average of 1.48 individual samples supporting one MI, ~24 % of MIs supported by at least two samples in the same population, and approximately 24 % of MIs are supported by different populations in the same ancestry category. Herein the results of MIs supported by multiple reads, individual samples, and populations suggest that our method is probably informative and reliable with an integrated view of individual samples. In addition, Additional file 1: Figure S2 shows that the length of MIs detected in 1KGP data is from 15 bp to 43 bp (mean length is 24 bp). As the majority of MIs concentrate within the size range from 18 bp to 31 bp, we assume that the incidence of MI would be higher in this size, which might be helpful for understanding the mechanism of MI. Furthermore, Additional file 1: Figure S3 shows the distribution of number of MIs across the human chromosomes, where the number of MIs generally has positive correlation with the length of chromosomes except chr11.

**Table 2:** Overview of MIs detected in 638 individual samples from 1KGP

population	sam-num	MI-num	MI-occ	mul-sup	read-num	occ/num	mul-sup/num	read/num
MXL	48	79	153	23	175	1.94	29.11 %	2.22
PUR	48	117	167	26	185	1.43	22.22 %	1.58
CLM	26	60	83	14	91	1.38	23.33 %	1.52
PEL	20	40	58	12	63	1.45	30.00 %	1.58
Total America	142	206	296	55	514	1.44	26.70 %	2.50
CDX	40	40	65	11	71	1.63	27.50 %	1.78
CHB	26	42	52	7	57	1.24	16.67 %	1.36
CHS	41	56	72	10	79	1.29	17.86 %	1.41
JPT	58	160	325	55	356	2.03	34.38 %	2.23
KHV	31	51	81	14	96	1.59	27.45 %	1.88
Total East Asia	196	262	349	53	659	1.33	20.23 %	2.52
GIH	11	22	29	6	34	1.32	27.27 %	1.55
Total South Asia	11	22	22	0	34	1.00	0.00 %	1.55
CEU	21	67	82	10	94	1.22	14.93 %	1.40
FIN	41	72	87	6	93	1.21	8.33 %	1.29
GBR	35	80	104	16	114	1.30	20.00 %	1.43
IBS	24	56	80	14	86	1.43	25.00 %	1.54
TSI	9	28	34	5	37	1.21	17.86 %	1.32
Total Europe	130	223	303	39	424	1.36	17.49 %	1.90
YRI	42	156	239	41	255	1.53	26.28 %	1.63
LWK	54	113	180	28	193	1.59	24.78 %	1.71
ACB	14	35	46	9	48	1.31	25.71 %	1.37
ASW	49	127	262	49	286	2.06	38.58 %	2.25
Total Africa	159	284	431	90	782	1.52	31.69 %	2.75

The “sam-num” column illustrates the number of samples for each category (either population or population group); the “MI-num” column illustrates the number of different MIs detected in each population or population group; the “MI-occ” column illustrates the sum of occurrences of MIs in each population or population group; the “read-num” column illustrates the number of reads supporting MIs. For the population lines, the “mul-sup” column illustrates the number of MIs supported by at least two individual samples (named “multiple samples supported MIs”) in one population, the “ooc/num” column illustrates the ratio of MI occurrence over MI number, which indicates the average number of individual samples supporting one MI in the same population, and the “mul-sup/num” column illustrates the ratio of multiple samples supported MIs over the number of all MIs. For the population group lines (which started with “Total”), the “mul-sup” column illustrates the number of MIs supported by at least two populations (named “multiple populations supported MIs”) in the same population group, the “ooc/num” column illustrates the ratio of MI occurrence over MI number, which indicates the average number of populations supporting one MI in the same population group, and the “mul-sup/num” column illustrates the ratio of multiple populations supported MIs over the total number of MIs. The last “read/num” column illustrates the ratio of the number of reads containing MIs over the number of MIs, which indicates the average number of reads supporting each MI

**Fig. 1:**
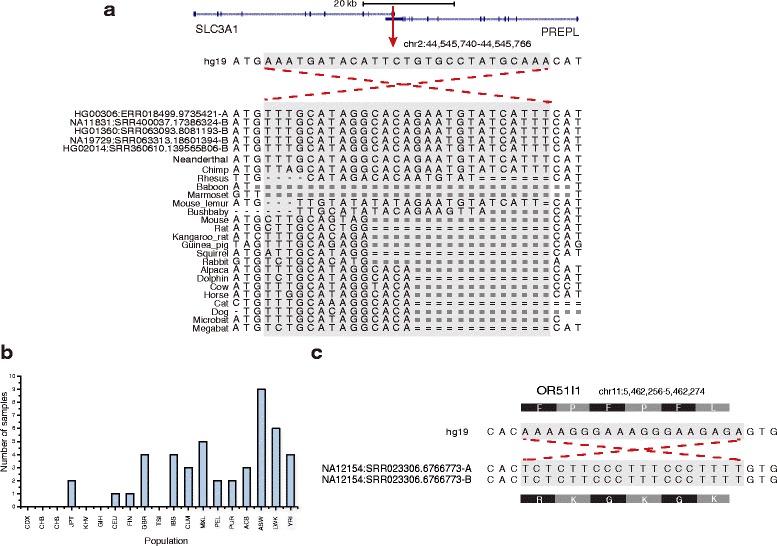
MI examples in 1KGP data. **a** shows one MI supported by 46 samples which overlap with 3’ UTR of gene *PREPL* and *SLC3A*; **b** shows the distribution of the MI supported by 46 samples in different populations; **c** shows one MI changing 6 amino acids of the exon in gene *OR51I1*

To focus on the exonic MIs detected, we checked 30 MIs overlapping with exons annotated by GENCODE. For instance, in Fig. 1a, an MI overlaps with the 3’ UTR of gene *PREPL* and gene *SLC3A1*, which both have strong correlation with Hypotonia-Cystinuria Syndrome [25, 26]. This MI is supported by 46 individual samples across different populations, including two samples in East Asian group, 10 samples in European group, 12 samples in Americas group, and 22 samples in African group (Fig. 1b). The chimpanzee sequence in this location is almost identical to the sequence of Neanderthal (Vi33.25 Sequence Reads) and the MI we found, which suggests that the reference human genome is inverted in this region as compared to the most recent common ancestor of the human population. However, this MI was reported as multiple nucleotide variation (GenBank:rs71416108) due to the poor understanding of MI [27]. Thus our identification of MI can be helpful for understanding genomic variants. Another MI changes 6 amino acids in the CDS region of gene *OR51I1* (Fig. 1c). These amino acids are located on the fourth transmembrane domain facing the less conserved extracellular side comparing to the intracellular side, which might cause severe influence.

### Application to CCLE lung squamous cell carcinoma data

In addition to the 1KGP data, cancer genome sequencing data are widely available. A full understanding of somatic alterations in cancer genomes is important to better understand the genetic basis for cancer development [28, 29]. Of the whole CCLE data, Lung Squamous Cell Carcinoma is a typical kind of lung cancer, which is a leading cause of death among all cancer patients [30]. In this work, we selected 14 WXS datasets of Lung Squamous Cell Carcinoma from CCLE to provide a proof-of-concept demonstration of the applicability of MID to cancer sequencing data (full sample list in Additional file 1).

**Fig. 2:**
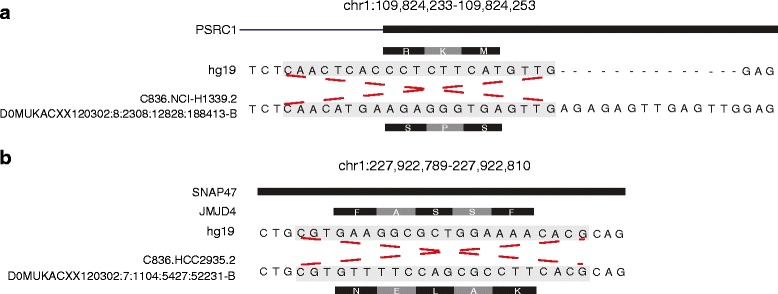
MI examples in Lung Squamous Cell Carcinoma WXS data from CCLE. **a** shows one MI breaking the edge of CDS region and changing 3 amino acids of gene *PSRC1*, as well as an insertion next to the MI; **b** shows one MI changing 5 amino acids of gene *JMJD4* and overlapping with 5’UTR of gene *SNAP47*

We found 12 MIs overlapping with 15 genes (Additional file 1: Table S2). To be more specific, seven MIs overlap with CDS regions, one MI overlaps with one gene in CDS regions and another gene in UTR regions, and the rest locate in the CDS nearby regions, which are annotated by GENCODE. Moreover, three MIs overlap with the proximal transcription factor binding sites (TFBS) annotated by ENCODE following ChIP peaks of transcription factors (more details can be found in Additional file 1: Table S2). We also found one MI within the 2,000 bp upstream region of gene *VPS54*. In Fig. 2a, we show that an MI breaks the edge of CDS region and changes three amino acids of gene *PSRC1*, which encodes a proline-rich protein that is a target for regulation by the tumor suppressor protein p53. In addition, Fig. 2b presents an MI changing five amino acids of gene *JMJD4* and overlapping with 5’UTR of gene *SNAP47*. While *JMJD4* is a member of JmjC-domain-only family, which only contains the JmjC domain, and plays an important role in demethylation and involves in cancer diagnosis [31]. The variants and expression bias of genes in this family are related to cancer regulations, thus the change made by MI in the *JMJD4* gene may help enrich the study of *JMJD4* and related cancer diagnosis.

## Discussion and conclusions

As presented above, upon test of the simulated data, MID has a steady performance of PPV for both low coverage and high coverage data, varying from 2X to 60X. Significantly, MID demonstrates high PPV in simulated dataset with low coverage 2–4X, which is the same as the coverage range used by low coverage samples from the 1KGP. In fact, the reason of our method showing stable performance on low coverage data is that the identification process is designed based on the target regions of read and reference sequence, regardless of read depth. Thus coverage bias has little influence in our method. By contrast, most existing tools require enough coverage to detect breakpoints, and only optimize good performance with high coverage data [4–10]. However, during our anchoring approach, in which the Bowtie program was called to make anchored alignment as preprocessing, higher coverage data can provide more detectable reads before identification process. Therefore we will have more reads containing possible MIs to be detected during identification, which means our strategy will benefit from higher coverage data. Therefore, although MID performs well in low coverage data, it can also benefit from data with higher coverage.

In fact, the pipeline of MID contains preprocessing and sequence mapping process. During the preprocessing of MID, we did anchored alignment by calling Bowtie. The reason we chose Bowtie [32] instead of the latest Bowtie2 [33] is that the length of paired-end reads (anchors) is shorter than 50 bp in ~100 bp NGS short reads, and Bowtie outperforms Bowtie2 in this size range, which is proved by our test and also claimed clearly in the tool clarification [33]. For sequence mapping, MID can handle small size MIs and MIs with multiple breakpoints, owing to the flexible segment mapping and scoring system during path finding. In existing tools such as BWA tool, although a number of discontinuous gaps and SNVs might be detected, it is extremely difficult to identify more complex SVs including inversions and duplications. In addition, more complicated scenario, i.e., MIs with multiple breakpoints within the read, would hardly be taken into consideration by these tools. MID uses flexible segment mapping on both strands based on *k*-mers, which is more suitable for MI detection, especially for dealing with small MIs and MIs with multiple breakpoints around. The scoring process also helps confirm the final path and distinguish incorrect matches, including palindromic sequences, and MIs as well.

In summary, we have developed a novel computational tool MID to identify MIs by mapping initially unmapped short reads back onto human genome sequence. What makes MID different from other SV detecting tools is that our approach is sensitive to very small size MIs, and capable of detecting MIs with multiple breakpoints in one read due to flexible segment mapping process, as well as scoring system in distinguishing MIs from palindromic sequence and other incorrect matches. The pipeline of MID can start from unmapped BAM files and find the optimum solution automatically rather than parameter changing. To our knowledge, MID is the first method that can efficiently and reliably identify MIs from unmapped short NGS reads. Moreover, MID is reliable on low coverage data, which is suitable for large-scale projects such as the 1KGP. We realize that the mechanism and the function of MI, as a kind of SV (as both germline and somatic alterations), are still poorly understood. Nevertheless, we expect that our tool would be useful to better understand MIs and their roles in genetic diversity and diseases. In conclusion, MID is suitable for large-scale short reads produced by present high-throughput sequencing technologies (e.g., Illumina), especially for low coverage data, and MID might have positive impact on identifying key genetic variants in human diseases with the further development of sequencing technologies.

## Methods

**Fig. 3:**
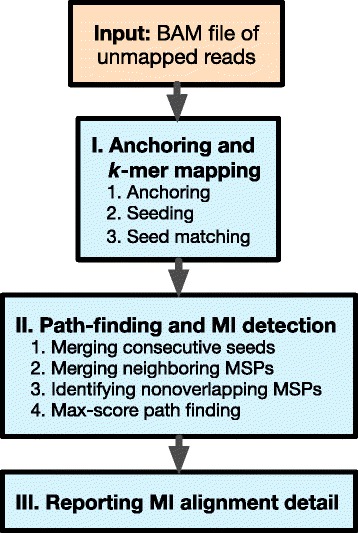
The workflow of the MID program

With an input of a BAM file containing unmapped short reads, MID takes three steps to report the output of a list of MIs, as well as detailed alignment information for each MI. The three main steps for identifying MIs are: (1) Create anchored alignment, which determines the corresponding region on the reference genome for the reads that might harbor MI. (2) Perform detailed alignment between the read and the genomic region identified in Step (1). (3) Report a list of MIs with additional information on how the MI-containing reads can be aligned. The workflow of MID is shown in Fig. 3.

### Anchoring and *k*-mer mapping

#### Anchoring

**Fig. 4:**
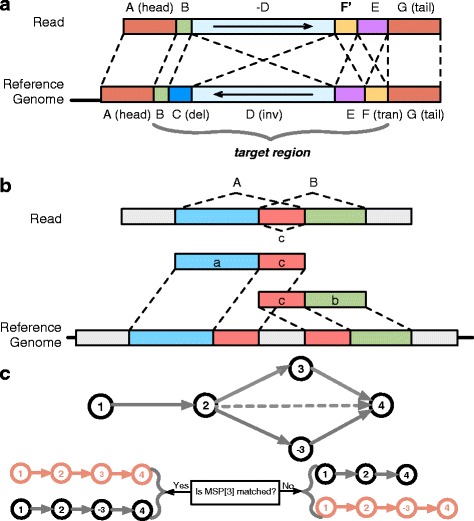
Methods overview of MID. **a** shows the construction of alignment regions. Head (*A*) and tail (*G*) substrings of read serve as a pair of anchors. Segment *C* in reference sequence denotes a deletion in read, segment *D* denotes an inversion in read and segment *F* denotes a translocation. A set of segment (*B, C, D, E, F*) in the reference sequence, as well as a set of segment (*B, −D, F’, E*) in the read sequence, form the target regions. **b** shows how to use a partition and recombine strategy to transfer the pair of overlapping MSs (*A, B*) to a set of non-overlapping MSs {(*A, b*), (*a, B*), (*a, b*)}. Segment *A* and *B* have overlapping region *c*, and segment *a* and *b* are generated by cutting region *c* off the original segment *A* and *B*. **c** shows the max-score path approach. The path starting with *MSP*_[1]_ and ending with *MSP*_[4]_ denotes a short read to be detected, and the path in red is the max-score path. *MSP*_[1]_, *MSP*_[2]_, *MSP*_[3]_, *MSP*_[4]_ are on the forward strand, and *MSP*_[−3]_ is on the reverse strand. MID starts with *MSP*_[1]_ and extends the path to *MSP*_[2]_, then if *MSP*_[3]_ and *MSP*_[−3]_ can both be matched, MID records both path candidates and ends with *MSP*_[4]_, therefore we have two path candidates {1, 2, 3, 4} and {1, 2, −3, 4}, then {1, 2, 3, 4} instead of {1, 2, −3, 4} will be chosen after scoring due to the reverse penalty for *MSP*_[−3]_ on the reverse strand. Otherwise if only *MSP*_[−3]_ is successfully matched, we have path candidates {1, 2, 4} and {1, 2, −3, 4}, after scoring {1, 2, −3, 4} would be chosen owing to the gap penalty, since path candidate {1, 2, 4} contains much more gaps. Therefore *MSP*_[−3]_ would be reported as detected MI

We provide an option of cutting size (0 bp as default) for both head and tail regions, which might be more error-prone, of each unmapped short read, and then select the new head and tail regions after cutting as “anchors”. As for short reads of ~100 bp, we set no cutting of two ends and select the head and tail regions of each unmapped short read as “anchors” (18 bp as default), afterwards we map the two anchors onto the reference genome as two ends of one paired-end read with a length of 18 bp using Bowtie [32] to find potential MI regions in the read (Fig. 4a). Moreover, for longer short reads (e.g., ~200 bp), we recommend choosing cutting size according to sequencing quality before selecting anchors. One mismatch on the anchors generated from a short read is allowed during mapping. Take the short read data of individual sample NA19213 as an example. The length of short reads in NA19213 is 76 bp, so the original distance between two anchors in the same read is 40 bp. Owing to the possible combination of variants including unbalanced variants such as small indels, insertions, deletions and duplications, we set the range of distance between two anchors to be in a range from 15 bp to 65 bp, which is denoted as the insert size for the paired-end read in Bowtie. If the distance is shorter than 15 bp caused by a possible deletion, then there is no need to consider the read since our algorithm aims to detect MIs longer than 15 bp. If the distance is longer than 65 bp caused by a possible insertion, we suppose this match could be redundant since we focus on MI detection and the inversion length in this read can be 40 bp at most. The length of anchors can be adjusted by users. However, we suggest a minimum of 10 bp set; otherwise the pair of anchors might not have unique alignment result in this step. This process significantly reduces the potential search space for MID.

#### Seeding

A *k*-mer extracted from the read is called a seed (14 bp as default). In addition to the head and tail anchors of a read, the middle part of the read is called “target region” (Fig. 4a). We then select target region of the read and extract consecutive seeds (step size 1 bp as default). We also extract consecutive seeds (step size 1 bp as default) from the reverse complement of the target region. These two groups of seeds are stored and operated separately for following steps.

#### Seed matching

The matching process is performed on the target regions both of the read and the reference sequence. After the anchoring process, we get the exact location of unmapped short read onto the reference sequence. We then select the target region of the initial read and its corresponding target region on the reference sequence. This seed-matching process can tolerate MIs with indels around the inversion breakpoints. We allow a maximum of *i* (2 as default) mismatches in a seed. In other words, if more than *i* mismatches occur within an interval of *k*, this *k*-mer (seed) will not be aligned. During seed matching, we compare each seed in the read target region to the reference target region and store all possible locations for each seed on the reference. To reduce the effect of a particular type of palindromic sequence tandem repeats (such as “AT”s or “GC”s) when matching the seed in the reverse complement to detect inversions, we discard the seeds with greater and equal to *n* (*n* = *L*/4, where *L* is the length of the substring sequence) “AT”s or “GC”s.

### Path finding and MI identification

This step aims to obtain the best path of *k*-mer alignment. MI within a read will correspond to a reversed sub-path.

#### Merging consecutive seeds

One seed can be matched to multiple locations on the reference sequence due to its short length. If the number of collinear consecutive seeds on the reference is larger than a threshold (5 as default), we consider this set of seeds as a “matching segment” (MS) and the “matching segment pair” (MSP) between the read and the reference. We might get different constructs of MSPs containing the same seeds, or one MS in the read that can be matched to different locations on the reference sequence, resulting in multiple MSPs for the same MS. The information of every possible MSP is recorded and will be further evaluated in the following steps.

#### Merging neighboring MSPs

While we set the threshold of *i* (2 as default) mismatches in one seed, if the SNVs occur at the edges of MSPs and a *k*-mer covering the SNVs reach the threshold of *i* mismatches, then this *k*-mer fails to be aligned. To overcome the problem caused by mismatches that is possibly caused by SNVs, we merge MSPs if the neighboring MSPs are in the same orientation and within the same given distance (3 bp as default) both on the read and on the reference sequence.

#### Identifying non-overlapping MSPs

MSPs may overlap with each other. Since we want to find paths constructed from non-overlapping MSPs, we take a partition-and-recombine strategy to make sure all the information carried by the MSPs is well kept (Fig. 4b). Before this step, MSPs in different orientations are stored and operated separately. We then put two separate sets of MSPs together and sort them by their locations on the reference sequence. Next, we find out each pair of overlapping MSPs and partition the overlapping subsequence to get a new set of non-overlapping MSPs pairs. We then recombine the overlapping subsequence with either MSP or neither of them. In Fig. 4b, for a pair of overlapping MSs (*A, B*) on the read, which has overlapping subsequence *c*, we partition *A* into *a* and *c*, and partition *B* into *b* and *c*, then we recombine the MSs as {(*A, b*), (*a, B*), (*a, b*)}. In this way, we change the former overlapping MS (*A, B*) into a set of non-overlapping MSs {(*A, b*), (*a, B*), (*a, b*)}. For overlapping MSPs, we use the partition-and-recombination strategy to check overlapping MSs on both read and reference sequence to get non-overlapping MSP set. This procedure guarantees the transformation of MSP is lossless, because we keep all the possible combinations in the non-overlapping MSP set. The resulting non-overlapping MSPs will be used in the subsequent path finding step.

#### Maximum-score path finding

We generate all combinations of non-overlapping MSPs as path candidates (Fig. 4c) and calculate the alignment score of each candidate to find the path with the maximum score by dynamic programming (see definitions of variables below). In dynamic programming, we define two kinds of score. One is for the MSP itself, which includes the score for matches and mismatches. The other is for the gap penalty between two neighboring MSPs in the path. We record the maximum score *T*_[*i*]_ of every path ending with *MSP*_[*i*]_ based on the maximum score of any sum of *T*_[*j*]_ and *GS*_[*j*,*i*]_ (1 < *j* < *i*). The complexity of our algorithm is *O*(*n*^2^), where *n* is the number of MSPs. In practice, ~85 % of *n* is smaller than 10. We consider match, mismatch, and gap in the similarity score [34]. Furthermore, for segments matched to the reverse strand, we use a penalty *R*_[*i*]_ for the potential inversion, which aims to reduce false positives caused by palindromic sequence and other incorrect matches. In fact, *MSP*_[*i*]_ of palindromic sequence can be matched to both strands and the one on the reverse strand would be added the penalty *R*_[*i*]_, which results in lower *F*_[*i*]_ compared with the same segment matched on the forward strand. Thus the final path finding based on maximum alignment score would select the match on the forward strand and avoid the influence of palindromic sequence.

The variables we used include:

- *n*: The number of MSPs.
- *M*_[*i*]_: The number of matches in *MSP*_[*i*]_.
- *MS*_[*i*]_: The number of mismatches in *MSP*_[*i*]_.
- *G*_[*i*,*j*]_: The number of gaps between *MSP*_[*i*]_ and *MSP*_[*j*]_.
- *R*_[*i*]_: The empirical reverse penalty for MSP based on our simulation, where one segment is the reverse complement of the other.
- *F*_[*i*]_: The score for *MSP*_[*i*]_.
- *G*_[*i*,*j*]_: The score for the gap penalty of *MSP*_[*i*]_ and *MSP*_[*j*]_. *GS*_[0,*i*]_ refers to the penalty of gaps from the first base of the sequence to the start of *MSP*_[*i*]_, and *GS*_[*i*,0]_ refers to the penalty of gaps from the end of *MSP*_[*i*]_ to the last base of the sequence.
- *GS*_[*j*,*i*]_: The score for the gap penalty of *MSP*_[*j*]_ and *MSP*_[*i*]_.
- *T*_[*i*]_: The maximum score of every path ending with *MSP*_[*i*]_.

We define:

- $$ {R}_{\left[i\right]}=3{\left({M}_{\left[i\right]}+M{S}_{\left[i\right]}\right)}^2 $$
- $$ {F}_{\left[i\right]}=\left\{\begin{array}{ll}100{M}_{\left[i\right]} - 100M{S}_{\left[i\right]}\hfill & \left(MS{P}_{\left[i\right]}\  on\  the\  forward\  strand\right)\hfill \\ {}100{M}_{\left[i\right]} - 100M{S}_{\left[i\right]}-{R}_{\left[i\right]}\hfill & \left(MS{P}_{\left[i\right]}\  on\  the\  reverse\  strand\right)\hfill \end{array}\right. $$
- $$ G{S}_{\left[i,j\right]}=\left\{\begin{array}{c}\hfill 0\kern9.75em \left(\mathrm{no}\ \mathrm{gap}\ \mathrm{between}\ MS{P}_{\left[i\right]}\ \mathrm{and}\ MS{P}_{\left[j\right]}\right)\hfill \\ {}\hfill - 400 - 30{G}_{\left[i,j\right]}\kern16em \left(\mathrm{else}\right)\hfill \end{array}\right. $$
- $$ {T}_{\left[i\right]}=\left\{\begin{array}{ll}G{S}_{\left[0,1\right]} + {F}_{\left[1\right]}\hfill & \kern0.84em \left(i=1\right)\hfill \\ {} \max \left(G{S}_{\left[0,i\right]},{T}_{\left[1\right]}+G{S}_{\left[1,i\right]},\dots, {T}_{\left[i-1\right]}+G{S}_{\left[i-1,i\right]}+{F}_{\left[i\right]}\right)\hfill & \left(1<i\le n\right)\hfill \end{array}\right. $$

Finally we select the maximum alignment score:

$$ \max \left({T}_{\left[i\right]}+G{S}_{\left[i,0\right]}\right), \left(1\le i\le n\right) $$

As in Fig. 4c, the path starting with *MSP*_[1]_ and ending with *MSP*_[4]_ denotes a short read to be detected, and the path in red is the max-score path. *MSP*_[1]_, *MSP*_[2]_, *MSP*_[3]_, *MSP*_[4]_ are on the forward strand, and *MSP*_[−3]_ is on the reverse strand. MID starts with *MSP*_[1]_ and extends the path to *MSP*_[2]_, then if *MSP*_[3]_ and *MSP*_[−3]_ can both be matched, MID records both path candidates and ends with *MSP*_[4]_. Therefore we have two path candidates as follows: {1, 2, 3, 4} and {1, 2, −3, 4}. After scoring, path candidate {1, 2, 3, 4} will be chosen due to the reverse penalty for *MSP*_[−3]_ on the reverse strand, which helps distinguish palindromic sequences. Otherwise if only *MSP*_[−3]_ is successfully matched, we have path candidates {1, 2, 4} and {1, 2, −3, 4}, after scoring {1, 2, −3, 4} would be chosen owing to the gap penalty, since path candidate {1, 2, 4} contains much more gaps. Therefore *MSP*_[−3]_ would be reported as detected MI.

In summary, MID is generated based on flexible segment mapping, including non-overlapping MSPs identification, which is capable of identifying very small size MIs, regardless of multiple breakpoints in one read, and scoring system which can distinguish false positives caused by palindromic sequence and other incorrect matches. In addition, although the parameter values are provided optionally, our test shows that the default option appears to generate the best overall performance for our algorithm.

## Additional file

Additional file 1:
**Supplementary figures, sample lists used by MID and tables containing detailed information of MIs detected by MID as well as their annotations.** (PDF 1727 kb)

## Abbreviations

1KGP: 1000 Genomes project
CCLE: Cancer cell line encyclopedia
CDS: Coding sequence
MI: Micro-inversion
MID: Micro-inversion detector
MS: Matching segment
MSP: Matching segment pair
NGS: Next-generation sequencing
PPV: Positive predictive value
SD: Standard deviation
SN: Sensitivity
SNV: Single nucleotide variation
SV: Structural variation
TFBS: Transcription factor binding site
UTR: Untranslated region
