# Supplementary material for: Identifying micro-inversions using high-throughput sequencing reads
Source: BMC Genomics. 2016 Jan 11;17(Suppl 1):4. doi: 10.1186/s12864-015-2305-7 (PMC4895285; doi:10.1186/s12864-015-2305-7)
Supplement: Additional file 1: — Supplementary figures, sample lists used by MID and tables containing detailed information of MIs detected by MID as well as their annotations. (PDF 1727 kb) [file 12864_2015_2305_MOESM1_ESM.pdf]

Supplementary Figures

Fig. S1. The MAF format of unmapped read containing MIs.

```
@SRR063108.1193133 (HG01516.IBS)
s              hg19.chr5  179256626  100  +  180915260  AAG~AGATAGTTTTTTTATCTTTCCTCTCCTCCTAATG~TAG

s @SRR063108.1193133              0   72  +        100  AAG~AGA----------------------------------------------------ATG~TAG

s @SRR063108.1193133             54   28  -        100  -------------TAGTTTTTTTATCTTTCCTCTCCTCCTA------------
```

The first line is the name of short read, the second line starting with “s” is the reference sequence of the read, and the third and fourth line are alignments on both forward and reverse strand. For the “s” lines, the first column “s” stands for the alignment lines, the second column shows the name of the reference chromosome or the name of the read, the third column stands for the starting point of the following sequence, the fourth column shows the length of the aligned sequence, the fifth column describes the strand to which the following sequence is aligned (“+” stands for the forward strand, while “-” stands for the reverse strand), the sixth column shows the size of the entire source sequence, and the last column shows the aligned sequence.

Fig. S2. Length distribution of MIs detected in the 1KGP data.

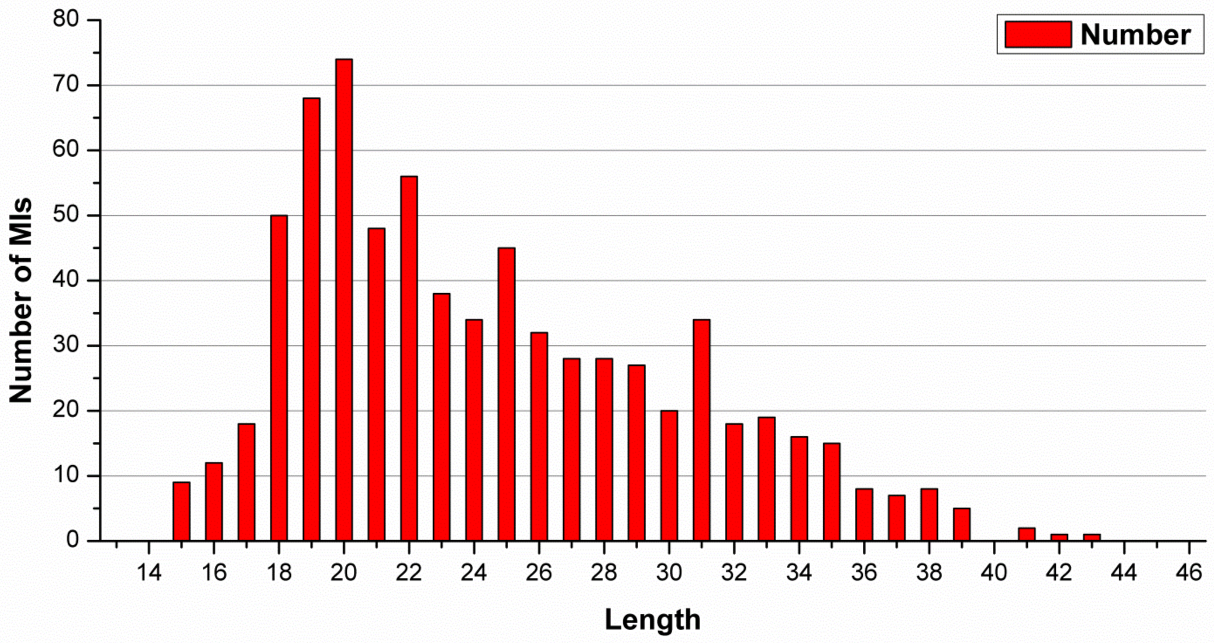

Fig. S3. The distribution of MIs from the 1KGP data across human chromosomes (based on hg19).

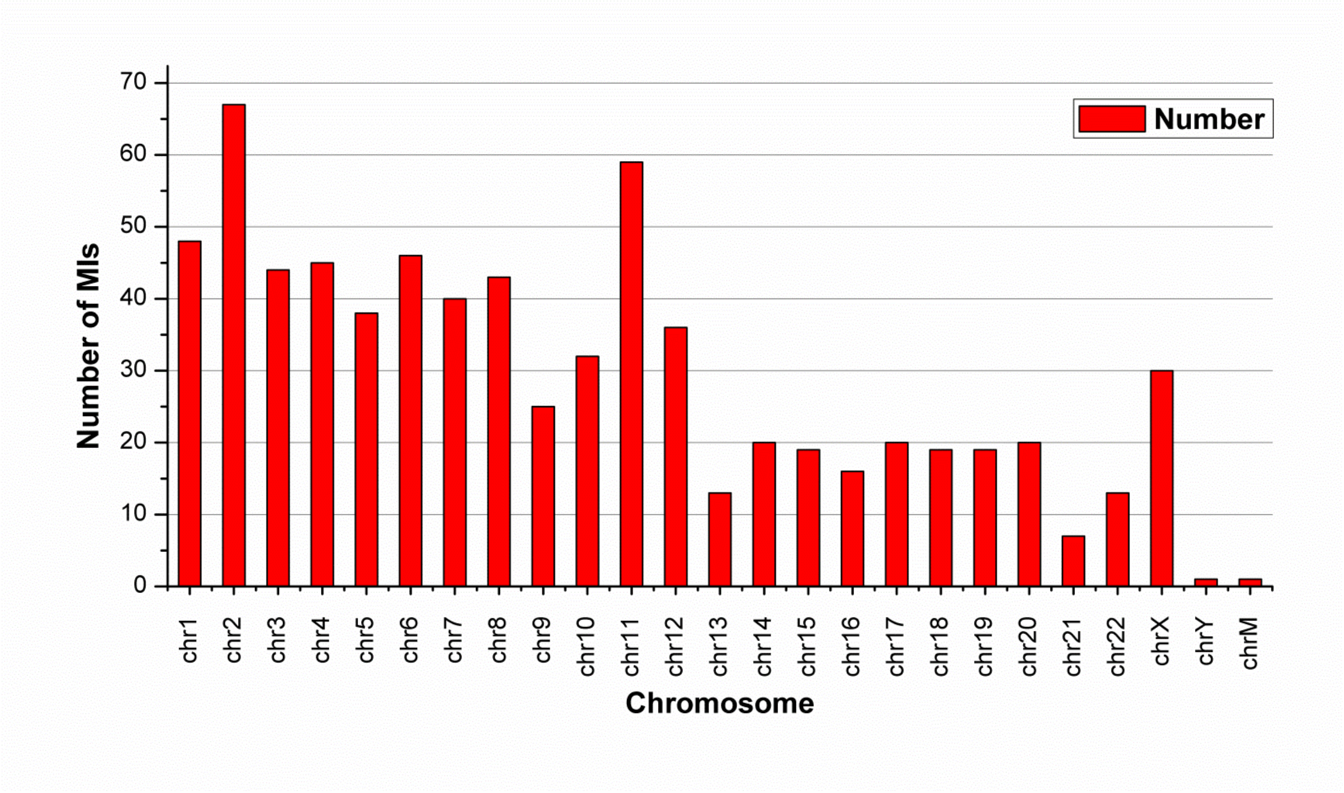

Sample List

The list of 638 samples from 1KGP, grouped by populations.

East Asia (CDX, CHB, CHS, JPT, and KHV)
CDX sample number: 40
HG01795, HG01796, HG00864, HG00879, HG01031, HG01797, HG01798, HG01799, HG01801, HG01802, HG01805, HG01806, HG01807, HG01810, HG01811, HG02373, HG02380, HG02392, HG02407, HG01812, HG01813, HG01815, HG02152, HG02164, HG02166, HG02178, HG02184, HG02186, HG02188, HG02375, HG02377, HG02382, HG02386, HG02387, HG02388, HG02390, HG02394, HG02395, HG02401, HG02402

CHB sample number: 26
NA18530, NA18533, NA18535, NA18537, NA18538, NA18539, NA18541, NA18553, NA18560, NA18565, NA18567, NA18572, NA18574, NA18602, NA18609, NA18611, NA18613, NA18615, NA18616, NA18617, NA18618, NA18619, NA18628, NA18630, NA18631, NA18634

CHS sample number: 41
HG00403, HG00404, HG00436, HG00449, HG00463, HG00464, HG00501, HG00533, HG00534, HG00537, HG00542, HG00560, HG00559, HG00566, HG00577, HG00578, HG00580, HG00581, HG00583, HG00592, HG00595, HG00611, HG00620, HG00625, HG00626, HG00629, HG00634, HG00656, HG00657, HG00662, HG00663, HG00671, HG00683, HG00684, HG00692, HG00693, HG00699, HG00702, HG00705, HG00707, HG00708

JPT sample number: 58
NA18950, NA18953, NA18960, NA18961, NA18963, NA18964, NA18968, NA18970, NA18971, NA18974, NA18975, NA18976, NA18981, NA18982, NA18983, NA18984, NA18986, NA18987, NA18988, NA18989, NA18990, NA18991, NA18999, NA19000, NA19003, NA19004, NA19005, NA19007, NA19009, NA19010, NA19012, NA19054, NA19055, NA19056, NA19057, NA19058, NA19059, NA19060, NA19062, NA19063, NA19064, NA19065, NA19066, NA19067, NA19068, NA19070, NA19072, NA19074, NA19076, NA19077, NA19078, NA19079, NA19080, NA19082, NA19083, NA19085, NA19087, NA19088

KHV sample number: 31
HG01599, HG01840, HG01842, HG01843, HG01844, HG01845, HG01846, HG01849, HG01850, HG01852, HG01855, HG01870, HG01871, HG01872, HG01873, HG01874, HG01878, HG02048, HG02057, HG02058, HG02060, HG02061, HG02064, HG02067, HG02069, HG02070, HG02073, HG02133, HG02134, HG02136, HG02137

South Asia (GIH)
GIH sample number: 11
NA21089, NA21090, NA21091, NA21092, NA21100, NA20867, NA20868, NA21098, NA21099, NA21102, NA21103

Europe (CEU, FIN, GBR, IBS, and TSI)
CEU sample number: 21
NA07048, NA11994, NA12005, NA12155, NA07346, NA11831, NA11832, NA11881, NA11992, NA12058, NA12154, NA12249, NA12272, NA12273, NA12275, NA12340, NA12341, NA12342, NA12399, NA12400, NA12718

FIN sample number: 41
HG00284, HG00285, HG00171, HG00173, HG00174, HG00176, HG00177, HG00179, HG00183, HG00186, HG00188, HG00189, HG00190, HG00266, HG00267, HG00269, HG00272, HG00274, HG00275, HG00276, HG00361, HG00277, HG00278, HG00280, HG00281, HG00306, HG00308, HG00310, HG00311, HG00313, HG00319, HG00320, HG00324, HG00325, HG00326, HG00327, HG00329, HG00336, HG00344, HG00357, HG00367

GBR sample number: 35
HG00119, HG00120, HG00160, HG00231, HG00233, HG00239, HG00242, HG00245, HG00246, HG00258, HG00262, HG00263, HG00264, HG00265, HG01334, HG01790, HG01791, HG00096, HG00103, HG00111, HG00112, HG00114, HG00116, HG00117, HG00122, HG00123, HG00124, HG00126, HG00127, HG00131, HG00133, HG00136, HG00137, HG00138, HG00142

IBS sample number: 24
HG01516, HG01519, HG01685, HG01694, HG01695, HG01756, HG01761, HG02232, HG02233, HG01501, HG01503, HG01506, HG01507, HG01510, HG01512, HG01513, HG01515, HG01518, HG01521, HG01522, HG01606, HG01607 HG01610, HG01669

TSI sample number: 9
NA20502, NA20540, NA20582, NA20586, NA20760, NA20769, NA20792, NA20796, NA20800

America (CLM, MXL, PEL, and PUR)
CLM sample number: 26
HG01112, HG01113, HG01124, HG01125, HG01136, HG01137, HG01140, HG01149, HG01250, HG01251, HG01253, HG01254, HG01341, HG01342, HG01350, HG01351, HG01353, HG01360, HG01366, HG01375, HG01378, HG01384, HG01438, HG01441, HG01455, HG01461

MXL sample number: 48
NA19648, NA19654, NA19649, NA19651, NA19652, NA19657, NA19663, NA19669, NA19678, NA19679, NA19684, NA19685, NA19719, NA19720, NA19747, NA19749, NA19750, NA19755, NA19756, NA19758, NA19759, NA19762, NA19770, NA19773, NA19774, NA19661, NA19676, NA19682, NA19722, NA19723, NA19725, NA19726, NA19728, NA19729, NA19731, NA19732, NA19746, NA19761, NA19776, NA19777, NA19779, NA19780, NA19782, NA19783, NA19785, NA19786, NA19788, NA19789

PUR sample number: 48
HG01069, HG01097, HG00638, HG01054, HG01082, HG01094, HG01098, HG01102, HG01107, HG00551, HG00553, HG00640, HG00737, HG00739, HG00740, HG01049, HG01051, HG01052, HG01060, HG01061, HG01067, HG01070, HG01072, HG01075, HG01079, HG01080, HG01101, HG01108, HG01110, HG01111, HG01167, HG01168, HG01170, HG01173, HG01174, HG01176, HG01177, HG01182, HG01183, HG01187, HG01190, HG01191, HG01197, HG01198, HG01204, HG01241, HG01242, HG01248

PEL sample number: 20
HG01577, HG01578, HG01917, HG01918, HG01920, HG01953, HG01954, HG01967, HG01970, HG01971, HG01973, HG01974, HG01976, HG01977, HG01982, HG02146, HG02291, HG02292, HG02298, HG02299

Africa (YRI, LWK, ASW, and ACB)
YRI sample number: 42
NA19213, NA19236, NA18486, NA18487, NA18488, NA18498, NA18504, NA18516, NA18520, NA18522, NA18853, NA18856, NA18867, NA18868, NA18871, NA18507, NA18874, NA18908, NA18910, NA18912, NA18917, NA18923, NA18924, NA18933, NA18934, NA19092, NA19116, NA19119, NA19130, NA19131, NA19152, NA19160, NA19171, NA19172, NA19197, NA19198, NA19200, NA19204, NA19223, NA19235, NA19247, NA19248

LWK sample number: 54

NA19332, NA19334, NA19346, NA19311, NA19313, NA19315, NA19316, NA19317, NA19318, NA19319, NA19321, NA19324, NA19327, NA19328, NA19331, NA19338, NA19347, NA19350, NA19372, NA19374, NA19375, NA19377, NA19385, NA19390, NA19391, NA19394, NA19397, NA19401, NA19403, NA19404, NA19429, NA19435, NA19438, NA19440, NA19443, NA19444, NA19445, NA19449, NA19451, NA19452, NA19453, NA19455, NA19456, NA19461, NA19462, NA19463, NA19466, NA19467, NA19469, NA19470, NA19471, NA19472, NA19473, NA19474

ASW sample number: 49

NA19625, NA19700, NA19704, NA19701, NA19703, NA19707, NA19711, NA19712, NA19713, NA19818, NA19819, NA19834, NA19835, NA19900, NA19901, NA19904, NA19908, NA19909, NA19914, NA19916, NA19917, NA19920, NA19921, NA19982, NA19985, NA20126, NA20127, NA20276, NA20278, NA20281, NA20282, NA20287, NA20289, NA20291, NA20294, NA20296, NA20299, NA20314, NA20317, NA20322, NA20332, NA20336, NA20340, NA20341, NA20342, NA20344, NA20346, NA20348, NA20356

ACB sample number: 14

HG01879, HG01880, HG01886, HG01896, HG01914, HG01915, HG01985, HG01986, HG02014, HG02051, HG02449, HG02470, HG02471, HG02489

The list of 14 samples applied from CCLE LUSC WXS data.

C836.Sq-1.1, C836.HCC2935.2, C836.COR-L24.1, C836.NCI-H1339.2, C836.NCI-H2286.1, C836.HCC-1438.4, C836.RS-5.1, C836.NCI-H1836.2, C836.DMS_79.3, C836.NCI-H1373.1, C836.NCI-H2073.1, C836.T3M-10.2, C836.NCI-H889.2, C836.SW_1573.1

Supplementary Tables

Table S1. MIs and annotations detected from the 1KGP data.

Name	Chr	Start	End	Len	MI	gene_name	gene_status	gene_type	exon	CDS	UTR	proximal_tfbs	distal_tfbs
@SRR026655.22810753-B	chr1	4929587	4929618	32	GTTCATGTGCACCGGGAGCCGCTAAATTCTTC	0	0	0	0	0	0	0	0
@ERR018442.20701969-A	chr1	8583719	8583738	20	AAGAAGTAACTCTAATGAGT	RERE	KNOWN	protein_coding	0	0	0	0	0
@ERR005750.12351363-B	chr1	9370691	9370718	28	TGCAGTGGTGCAATCACAACTCACTGCA	SPSB1	KNOWN	protein_coding	0	0	0	0	0
@SRR005971.4716312-B	chr1	11545061	11545087	27	GTTTTTGCCATCACTTTAAATGGCAAA	PTCHD2	KNOWN	protein_coding	0	0	0	0	0
@SRR359061.75479771-A	chr1	28202679	28202706	28	CACTAAGGGAGTTGGGATTCCCTTAGTG	THEMIS2	KNOWN	protein_coding	0	0	0	0	0
@SRR014630.1670073-A	chr1	28681815	28681831	17	AAGAGTGACAGTCAAGC	0	0	0	0	0	0	0	0
@SRR037777.13792296	chr1	30569420	30569455	36	CCCAGCCCAGCCCTATCATGGCATTAGGGGCTGGGC	0	0	0	0	0	0	0	0
@SRR023860.9874067-A	chr1	37529399	37529418	20	GTGAGCTGAGATTGTGCCAC	0	0	0	0	0	0	0	0
@SRR029934.12490023-B	chr1	45739850	45739880	31	TTAAAGCCTCTCTCCTCTTTCTTGTCTGTAA	ZSWIM5	KNOWN	protein_coding	0	0	0	0	0
@ERR018540.18622273-A	chr1	48823569	48823597	29	TTAGTTTTGAATTTACCTATACAAAACTA	SPATA6	KNOWN	protein_coding	0	0	0	0	0
@ERR020286.70280801	chr1	58225718	58225742	25	TCTCCACAAACTTCATTTGTGGAGA	DAB1	KNOWN	protein_coding	0	0	0	0	0
@ERR013126.4333632-B	chr1	58824966	58824986	21	TTTCATTGATTTACAAAGTAA	DAB1	KNOWN	protein_coding	0	0	0	0	0
@SRR019044.4010134	chr1	68552098	68552135	38	AAGAGATAGAGCATTGTGGTGAAGAGTTTCTATCACTT	GNG12-AS1	NOVEL	antisense	0	0	0	0	0
@ERR009399.17541402-A	chr1	73125489	73125507	19	TATTGTTCTTAGTAACATA	0	0	0	0	0	0	0	0
@ERR019895.13490778-B	chr1	76211938	76211965	28	ACTTATCCTACCAAAAGGTTGCTGTGAG	ACADM	KNOWN	protein_coding	0	0	0	0	0
@SRR062563.24415412-B	chr1	80521458	80521482	25	GGAATATCCTTTTTAGGTAATCTTT	0	0	0	0	0	0	0	0
@ERR022460.57946695-A	chr1	82212687	82212706	20	TGTGTACATATGTGTGTGTA	LPHN2	KNOWN	protein_coding	0	0	0	0	0
@ERR018440.59322289-A	chr1	82446407	82446437	31	AGAGAGAGATGCTAAAGATTAGCTCTCTCTC	LPHN2	KNOWN	protein_coding	0	0	0	0	0
@ERR018434.40374175-B	chr1	83129955	83129976	22	TTGTTGTACATGTTACATAAAA	0	0	0	0	0	0	0	0
@SRR038712.11521918	chr1	87251955	87251981	27	TTGTTAACATTATTGTTTTAGCAATAT	0	0	0	0	0	0	0	0
@SRR061611.4550448-A	chr1	89233690	89233725	36	TTATTTAAGCATCTCTATTTTTAATCACTAAGGCAA	PKN2	KNOWN	protein_coding	0	0	0	0	0
@ERR009321.19027993-B	chr1	96250154	96250177	24	TCACCAGAGAGAGACAAAGGGTGA	0	0	0	0	0	0	0	0

@ERR019895.24443627-B	chr1	97401135	97401160	26	CATACACACACATACACACG TGTATA	AL592205.2	NOVEL	miRNA	1	0	0	0	0
@ERR020261.76850904-A	chr1	101433164	101433183	20	GTTAGTCGTACCACATAGGT	SLC30A7	KNOWN	protein_coding	0	0	0	0	0
@ERR015510.15236622-B	chr1	101622622	101622646	25	TCCAGATACCTATAACAGTCT GGAT	0	0	0	0	0	0	0	0
@SRR022682.6058465-B	chr1	106945465	106945489	25	GTCTCTAAGAACATTCTGCTA GTGA	0	0	0	0	0	0	0	0
@ERR044627.100726496-A	chr1	108324757	108324779	23	TATTTTACTTGTTATTAGCTT GT	VAV3	KNOWN	protein_coding	0	0	0	0	ARI D3A, CTC F
@ERR043026.11607799-B	chr1	108344208	108344231	24	ACCACTCAATAGTCTATGAGT GGT	VAV3	KNOWN	protein_coding	0	0	0	0	0
@SRR014728.1301254-A	chr1	111023187	111023206	20	CACACCGAGGGAGGCCATCT	0	0	0	0	0	0	0	0
@SRR027538.4084564	chr1	118261195	118261217	23	AGCATTAGGAGATACACCTA ATG	0	0	0	0	0	0	0	0
@ERR022462.64254411-A	chr1	145448182	145448202	21	TGAACCCAGGAGGAGGAGGT T	0	0	0	0	0	0	0	0
@SRR111966.8017300	chr1	162885157	162885189	33	CCGGGGTACATATGTAGGTA CCATGTACCCCTG	0	0	0	0	0	0	0	0
@SRR360541.16945369-B	chr1	166639054	166639073	20	TTTAAGGGCAAACACTTAAA	FMO10P	KNOWN	pseudogene	1	0	0	0	0
@ERR019895.18849516-A	chr1	173992271	173992293	23	TCTCTCTCCTCTATTCTAGAT GA	0	0	0	0	0	0	CHD1,T FAP2A	0
@ERR052929.6785190	chr1	175467295	175467313	19	CATTTCTGCTACCAGGAAT	TNR	KNOWN	protein_coding	0	0	0	0	multi ple
@SRR029836.4404478	chr1	177614384	177614421	38	TTTGAGCTTCTTAAAGGAACA AATAAACACAAGTCAAA	0	0	0	0	0	0	0	0
@ERR042514.48911601	chr1	192240100	192240122	23	TCTATTTTCAGCTTTAAAATA GA	0	0	0	0	0	0	0	0
@SRR006285.4182375-A	chr1	195811657	195811678	22	AAAAATCCAAACACACAGAC AC	0	0	0	0	0	0	0	0
@SRR189829.23979121-B	chr1	201236189	201236208	20	ATCCAAATTTGTGTGTCTGT	0	0	0	0	0	0	0	multi ple
@ERR016342.18684136-A	chr1	211464666	211464690	25	TTTGAGCATTATGTCAGTGCT CAAA	RCOR3	KNOWN	protein_coding	0	0	0	0	0
@SRR031345.21658266	chr1	217796823	217796850	28	TAAGAAGTAAATTACTATTA CTAATAAA	GPATCH2	KNOWN	protein_coding	0	0	0	0	0
@SRR029899.9590035	chr1	222507802	222507823	22	AGGAAAATGGTACAAATTTG GT	RP11-400N13 .1	NOVEL	lincRNA	0	0	0	0	0
@SRR360610.29828561-A	chr1	223947588	223947613	26	GTGGGAACCGTTGAGATGGT TCCCAC	CAPN2	KNOWN	protein_coding	0	0	0	0	0
@SRR020479.6314510	chr1	225781831	225781859	29	TTGAATCTAGCAGTGTTCCAG AGAAGCAA	ENAH	KNOWN	protein_coding	0	0	0	0	0
@SRR064187.64080025	chr1	247989262	247989281	20	TATATGTGTGTGTGTATATA	0	0	0	0	0	0	0	0
@SRR031345.18166666	chr1	248607338	248607368	31	AAGCAGAAAACCCATACCCT TTTTCAGCCTT	0	0	0	0	0	0	0	0
@SRR064388.51321887-A	chr1	248621397	248621431	35	ATAAGCTCCAATTTTAAAATA GTTAAACCACTTAT	0	0	0	0	0	0	0	0
@SRR015990.2109882	chr1	248775495	248775531	37	AAATAGAGGAGCCGTGGGGC TGAACAAGGGCCTATCT	0	0	0	0	0	0	0	CTC F
@ERR050171.10505929	chr10	2538793	2538814	22	TATATACACACACACACACA CA	0	0	0	0	0	0	0	0

@SRR014131.2600132	chr10	3173061	3173093	33	TTTGGCTCAGAAACAAGTAA CATCCTAACAGAT	PFKP	KNOWN	protein_coding	0	0	0	0	0
@SRR014133.6185750	chr10	3176609	3176639	31	ATACAGTAACACTTTGCTGTT AAATAGAAAT	PFKP	KNOWN	protein_coding	0	0	0	0	0
@SRR014202.4151223	chr10	3790572	3790593	22	TCTTTTTTTTTTTTTTTTTTTT	0	0	0	0	0	0	0	0
@ERR018546.1907483-A	chr10	7139997	7140023	27	GACCCCATCATCTCTCTGTTT GGGGTC	0	0	0	0	0	0	0	0
@SRR350138.3998458-A	chr10	7422616	7422632	17	TGCCCTTAAAAAAAAAA	SFMBT2	KNOWN	protein_coding	0	0	0	0	0
@ERR018545.6791411-B	chr10	10616722	10616751	30	TGCAGTGAGCAAAGATCATG CCACTGCACT	0	0	0	0	0	0	0	0
@ERR009381.10474299-B	chr10	13280521	13280545	25	GTCTAAGCCTCAGCTTAGAC ATGTC	0	0	0	0	0	0	0	0
@SRR032770.13362519-A	chr10	17022109	17022128	20	TTTTATTTGCATTTCTCTTA	CUBN	KNOWN	protein_coding	0	0	0	0	0
@SRR014204.956355	chr10	25513662	25513691	30	TGCAAAAATTGGTAGCAAAC ATAGTTGACA	GPR158	KNOWN	protein_coding	0	0	0	0	0
@SRR111942.85916919-B	chr10	33392142	33392167	26	TGTTTGGCGCTGAGAAGAGC CAAACA	RP11-342D11 .3	NOVEL	lincRNA	0	0	0	0	0
@SRR061650.2450562-A	chr10	33418396	33418416	21	CTACAGCCATTCAGGGGACT G	0	0	0	0	0	0	0	0
@SRR023394.10777880-A	chr10	34977704	34977723	20	CAAAATTAGCCCTAATTTAC	PARD3	KNOWN	protein_coding	0	0	0	0	0
@ERR019495.2468727-A	chr10	38775646	38775666	21	TGGAATCAATTCGAGTGCAA T	0	0	0	0	0	0	0	0
@ERR018547.7862531-A	chr10	50074823	50074844	22	GTGTCACCTATGCTGACACAC T	WDFY4	KNOWN	protein_coding	0	0	0	0	0
@ERR015529.1148096-B	chr10	52579310	52579326	17	GCGAGTGGGGACTGGGG	A1CF	KNOWN	protein_coding	0	0	0	0	0
@ERR020234.63099714-B	chr10	57531391	57531413	23	TATACACACAAATACATACC TAA	0	0	0	0	0	0	0	0
@ERR042949.47604594	chr10	61808458	61808486	29	TGGGCATTCCACATGCACAA ACTCAACCA	ANK3	KNOWN	protein_coding	0	0	0	0	0
@ERR009411.12066175-B	chr10	66900159	66900197	39	TATGAACCAGGAAGTAGTCT CTCATCAAACTCCGAATCT	0	0	0	0	0	0	0	0
@ERR018555.7204989-B	chr10	86654951	86654973	23	CTTTTTTTAAAAAAAAAAAA AAG	0	0	0	0	0	0	0	0
@ERR020286.20786526-B	chr10	86681736	86681755	20	AGTCTTGAGAGGTCAGGACT	0	0	0	0	0	0	0	0
@SRR063110.1705704-B	chr10	90454935	90454954	20	TATATGTGTGTGTGTATATA	0	0	0	0	0	0	0	0
@SRR360545.2238532-B	chr10	95080094	95080123	30	ATGACTTATAGTTGTGAGGA CTAAACTCAT	MYOF	KNOWN	protein_coding	0	0	0	0	0
@SRR032291.18820909-B	chr10	101016126	101016148	23	TGTAGGGGTGGGTTGCCCCT ACA	0	0	0	0	0	0	0	0
@SRR006185.307680	chr10	107864674	107864707	34	TGTTGGGGGGTAAAGTAGGG AATCCCCTTACACA	0	0	0	0	0	0	0	0
@SRR014128.1713431	chr10	108781289	108781305	17	AGTCTTTTTTTTTTTTT	SORCS1	KNOWN	protein_coding	0	0	0	0	0
@SRR065209.11029210-A	chr10	109621079	109621113	35	TAAATAAAATAGTGTAGATA GGGGTCTATCTTATC	RP11-215N21 .1	NOVEL	lincRNA	0	0	0	0	0
@SRR189830.107073385-A	chr10	115419260	115419286	27	TCCCCAGTGGTAGATTTGCAC TGGGGA	NRAP	KNOWN	protein_coding	0	0	0	0	0
@ERR019895.7829994-A	chr10	122778190	122778223	34	AGGAATTTACAGTTTTACTAT CTAGCATCTGCCT	0	0	0	0	0	0	0	0
@ERR005768.155865	chr10	125755331	125755350	20	GCCATGGGGCTCCTCCCAGG	0	0	0	0	0	0	0	0

@SRR062626.20344751-A	chr10	127604509	127604532	24	TGCTAATCTCAAGCAGTTATTACA	FANK1	KNOWN	protein_coding	0	0	0	0	0
@SRR064192.59223520	chr10	129153410	129153427	18	TACACTGCCACCAGGTGG	DOCK1	KNOWN	protein_coding	0	0	0	0	CTCF,RAD21,SMC3
@ERR012147.9206472-A	chr11	4538979	4539001	23	AAACAAGCCCATGCACATTTCAA	0	0	0	0	0	0	0	0
@SRR031308.11611170	chr11	5360622	5360649	28	GTTAGGAAAAGAAGCCTATTTATAACAC	HBG2	KNOWN	protein_coding	0	0	0	0	0
@SRR031308.11611170	chr11	5360622	5360649	28	GTTAGGAAAAGAAGCCTATTTATAACAC	HBE1	KNOWN	protein_coding	0	0	0	0	0
@SRR031308.11611170	chr11	5360622	5360649	28	GTTAGGAAAAGAAGCCTATTTATAACAC	AC104389.28	NOVEL	processed_transcript	0	0	0	0	0
@SRR360588.30227561-A	chr11	5390369	5390399	31	TGAATGCCTATTTTTAAAGACTTCATCCTTT	HBG2	KNOWN	protein_coding	0	0	0	0	0
@SRR360588.30227561-A	chr11	5390369	5390399	31	TGAATGCCTATTTTTAAAGACTTCATCCTTT	HBE1	KNOWN	protein_coding	0	0	0	0	0
@SRR360588.30227561-A	chr11	5390369	5390399	31	TGAATGCCTATTTTTAAAGACTTCATCCTTT	AC104389.28	NOVEL	processed_transcript	0	0	0	0	0
@SRR023306.6766773-B	chr11	5462245	5462283	39	GTTTCACCACTCTCTTCCCTTTCCCTTTTGTGGTGAAAC	HBG2	KNOWN	protein_coding	0	0	0	0	0
@SRR023306.6766773-B	chr11	5462245	5462283	39	GTTTCACCACTCTCTTCCCTTTCCCTTTTGTGGTGAAAC	HBE1	KNOWN	protein_coding	0	0	0	0	0
@SRR023306.6766773-B	chr11	5462245	5462283	39	GTTTCACCACTCTCTTCCCTTTCCCTTTTGTGGTGAAAC	AC104389.28	NOVEL	processed_transcript	0	0	0	0	0
@SRR023306.6766773-B	chr11	5462245	5462283	39	GTTTCACCACTCTCTTCCCTTTCCCTTTTGTGGTGAAAC	OR51I1	KNOWN	protein_coding	1	1	0	0	0
@SRR360610.93665891	chr11	6838450	6838469	20	AAACTCATCTTGGCTAGCAG	0	0	0	0	0	0	0	0
@SRR190849.39303371	chr11	7779543	7779570	28	TTTACATAAAATTGTAAAATTAAAAACA	RP11-35J10.5	NOVEL	lincRNA	0	0	0	0	0
@SRR190849.39303371	chr11	7779543	7779570	28	TTTACATAAAATTGTAAAATTAAAAACA	RP11-494M8.4	NOVEL	sense_overlapping	0	0	0	0	0
@SRR189830.121735803	chr11	9705131	9705154	24	ACACTTTTGGATTCCTAACAGTGT	SWAP70	KNOWN	protein_coding	0	0	0	0	SPI1
@SRR063266.8502584-A	chr11	11294503	11294535	33	TCTTGCATTTTCTCAGGCAATTTATTGAAAAAA	GALNT18	KNOWN	protein_coding	0	0	0	0	0
@SRR189830.40883302	chr11	14696102	14696129	28	AGAAGGAGGAGACAGCAAATCACTTATA	PDE3B	KNOWN	protein_coding	0	0	0	0	0
@ERR042971.42665675-A	chr11	16733622	16733641	20	TGTGTGTGTGTGTGTGTGTG	SOX6	KNOWN	protein_coding	0	0	0	0	0
@ERR042971.42665675-A	chr11	16733622	16733641	20	TGTGTGTGTGTGTGTGTGTG	C11orf58	KNOWN	protein_coding	0	0	0	0	0
@ERR042971.42665675-A	chr11	16733622	16733641	20	TGTGTGTGTGTGTGTGTGTG	SOX6	KNOWN	protein_coding	0	0	0	0	0
@ERR042971.42665675-A	chr11	16733622	16733641	20	TGTGTGTGTGTGTGTGTGTG	C11orf58	KNOWN	protein_coding	0	0	0	0	0
@ERR042971.42665675-A	chr11	16733647	16733665	19	TGTGTGTGTGTGTGTGTAT	SOX6	KNOWN	protein_coding	0	0	0	0	0
@ERR042971.42665675-A	chr11	16733647	16733665	19	TGTGTGTGTGTGTGTGTAT	C11orf58	KNOWN	protein_coding	0	0	0	0	0
@ERR042971.42665675-A	chr11	16733647	16733665	19	TGTGTGTGTGTGTGTGTAT	SOX6	KNOWN	protein_coding	0	0	0	0	0
@ERR042971.42665675-A	chr11	16733647	16733665	19	TGTGTGTGTGTGTGTGTAT	C11orf58	KNOWN	protein_coding	0	0	0	0	0
@SRR061620.5651977-B	chr11	18075842	18075860	19	TATACTATGTAAGTACATA	0	0	0	0	0	0	0	0

@SRR032231.8220550-B	chr11	19038027	19038046	20	CCGGTACTGTGCCAGAACAG	0	0	0	0	0	0	0	0
@SRR061612.5745603	chr11	20525940	20525966	27	TGTAAATCTTGCCATTTTGTT CTTTTT	PRMT3	KNOWN	protein_coding	0	0	0	0	0
@ERR015478.16029986-B	chr11	21148300	21148330	31	CCCAGTCTGAACACTGTCTAC ACAGCCTGGG	NELL1	KNOWN	protein_coding	0	0	0	0	0
@SRR189827.23812313	chr11	23266296	23266314	19	GATTTTTATTTTCCATTAA	0	0	0	0	0	0	0	0
@SRR034577.6767312-B	chr11	23968144	23968180	37	GAATCTTTGCATTTTTAGTAT TTTTACCTTGTGTCTC	0	0	0	0	0	0	0	0
@SRR360149.131581843	chr11	24089596	24089617	22	TTCTATCTGAGATGAGCGAAT A	0	0	0	0	0	0	0	0
@ERR020255.90769480	chr11	24103081	24103098	18	TACTTTCAGAAAAGTATT	0	0	0	0	0	0	0	0
@ERR022460.59159555-A	chr11	25119935	25119951	17	CACACACACACACACAC	0	0	0	0	0	0	0	0
@SRR062617.6967523-A	chr11	25923163	25923181	19	TACGTGTATACGTGTACAT	0	0	0	0	0	0	0	0
@ERR012115.26091225-B	chr11	25923187	25923202	16	GTATACGTGTATACGT	0	0	0	0	0	0	0	0
@SRR069535.61113358-A	chr11	32462270	32462294	25	TATTCAGTTTGAAAGATAATA CTGA	WT1-AS	KNOWN	antisense	1	0	0	0	0
@SRR069535.39839845	chr11	34269643	34269660	18	TTTTTTTTTTTTTTTTTT	ABTB2	KNOWN	protein_coding	0	0	0	0	0
@ERR022471.667508-B	chr11	37786876	37786903	28	TGTTTCACTACAAAAGCTTAA TGAAACA	0	0	0	0	0	0	0	0
@ERR016243.3931015	chr11	39568160	39568195	36	TAAAGAGTCTTATATTTTTTC TAAAAAAAGGCTTTA	0	0	0	0	0	0	0	0
@ERR009322.13436939-A	chr11	39752386	39752407	22	TATATACACACACACACATA TA	AC027806.1	NOVEL	miRNA	1	0	0	0	0
@ERR022464.60462969-B	chr11	44600552	44600578	27	GCCAGACAGAAGGGACCTGA CCTCAGC	CD82	KNOWN	protein_coding	0	0	0	0	0
@SRR190851.112652402	chr11	46691422	46691444	23	AGCTTTCAATCTCCTCTGCCC AG	ATG13	KNOWN	protein_coding	0	0	0	0	0
@ERR050128.20460792-B	chr11	46783980	46784005	26	GTGATACAGGCACCACTCAG TATCAC	CKAP5	KNOWN	protein_coding	0	0	0	0	0
@ERR050128.20460792-B	chr11	46783980	46784005	26	GTGATACAGGCACCACTCAG TATCAC	SNORD67	KNOWN	snoRNA	1	0	0	0	0
@ERR050119.12887258	chr11	57088749	57088770	22	TTGTTTGACTTGGGTCAATAG T	TNKS1BP1	KNOWN	protein_coding	0	0	0	CTCF,FOXA1,FOXA2,HDAC2,MYBL2	0
@SRR111942.79554812	chr11	57099809	57099834	26	TTTCCTACCACTGTCCAGGCA GGAGA	SSRP1	KNOWN	protein_coding	0	0	0	0	0
@SRR023393.6058459-A	chr11	57104787	57104804	18	TTATTTTTTTTTTTTTAT	0	0	0	0	0	0	0	0
@ERR009282.8324529-A	chr11	62865228	62865248	21	CTTTGTAGGGACATGGATGA A	SLC22A24	KNOWN	protein_coding	0	0	0	0	0
@ERR042518.51188066	chr11	65372368	65372392	25	AGCTCAACACTGCCCAGCTC AGTGT	MAP3K11	KNOWN	protein_coding	0	0	0	0	0
@SRR017209.16659196-A	chr11	70056326	70056344	19	GAAGCTTCTAAGAAGGCTC	0	0	0	0	0	0	0	POLR2A
@ERR034778.12623508	chr11	72733493	72733515	23	TTGCATTTATGTAGGAGAGC AAT	FCHSD2	KNOWN	protein_coding	0	0	0	0	0
@SRR032163.3573512	chr11	74219329	74219355	27	CTACTTCAATTCCTGCCACAA TGAAAG	POLD3	KNOWN	protein_coding	0	0	0	0	0

@ERR019907.29594998	chr11	75050248	75050268	21	GGAGACCCTGCAGAGATCTGA	ARRB1	KNOWN	protein_coding	0	0	0	0	0
@SRR111943.64586539	chr11	77122301	77122321	21	GCTTTTCCAGACTGGCANACC	PAK1	KNOWN	protein_coding	0	0	0	0	0
@ERR022462.40229707	chr11	77193521	77193551	31	AAAGTATATTGAAGGCTTCATCTTACTTTAG	0	0	0	0	0	0	0	0
@ERR022463.37875002	chr11	78211249	78211281	33	TTTCAGCTTGGGTCTTTAGACTACATGTTCAAA	NARS2	KNOWN	protein_coding	0	0	0	0	FOS
@ERR015744.18017900-B	chr11	79364801	79364824	24	TAGGAAATCTTGCTTATAGACCTG	0	0	0	0	0	0	0	0
@ERR015741.1022820	chr11	83521348	83521377	30	ACAATTTTCTTTCTTTAGGGAAATACTTGT	DLG2	KNOWN	protein_coding	0	0	0	0	0
@SRR015518.14538085	chr11	87185498	87185521	24	GTTTTTTCCATTACTTTAAATGAT	0	0	0	0	0	0	0	0
@ERR018472.77923228-A	chr11	88815364	88815387	24	TATGTATGTGTGTGTGTGTATATA	0	0	0	0	0	0	0	0
@ERR045708.39226951	chr11	89331146	89331165	20	ATTTCTAGGTANATTTATAC	0	0	0	0	0	0	0	0
@SRR189816.2535252	chr11	93382342	93382376	35	AAGTATTGACAAAACCTATCAACTTTGTCAATACT	0	0	0	0	0	0	0	0
@ERR015743.1173139	chr11	99092709	99092736	28	GTATGTGTATCTTTTTATCACTTACATA	CNTN5	KNOWN	protein_coding	0	0	0	0	0
@SRR015471.8504063	chr11	100739808	100739839	32	CCAACACAGATTTGTTGTTGTTGCATCCGAGC	ARHGAP42	KNOWN	protein_coding	0	0	0	0	0
@ERR018539.7673926-A	chr11	101944172	101944194	23	GGCACTGTCCCCCATCCAGTGCC	C11orf70	KNOWN	protein_coding	0	0	0	0	0
@SRR016225.11603779-B	chr11	114352745	114352766	22	ACATTTCAGTTGAAAGGTGGAT	0	0	0	0	0	0	0	0
@ERR020275.69739578	chr11	114675679	114675704	26	GAGAAAACATCCCCTGGGAGTTTCAT	0	0	0	0	0	0	0	0
@ERR018528.4672963-A	chr11	120058121	120058152	32	AGTTTGCTGGAATTCGGAGCACGTTGGTGTAC	0	0	0	0	0	0	FOXA1, FOXP2,SP1,WRNIP1,YY1	0
@ERR019898.6768030-B	chr11	120672596	120672620	25	CTGCAGCCCTCCACAGCTCTGGGAC	GRIK4	KNOWN	protein_coding	0	0	0	0	GATA3,TRIM28
@ERR013153.4945126	chr11	121787570	121787603	34	TACCACATTCTGACTATTGAACATGTGTGTGGTA	0	0	0	0	0	0	0	0
@ERR015759.4932650	chr11	124889150	124889178	29	TACTTTATCCATTAAGAGGAGATAAAGTA	CCDC15	KNOWN	protein_coding	0	0	0	0	0
@SRR350142.141748640-A	chr11	125896835	125896852	18	AGAGTTAACGTTGACTCT	CDON	KNOWN	protein_coding	0	0	0	0	FOXA1,FOXA2
@ERR022463.60458161-B	chr11	132245950	132245970	21	CACACATCCCCATTCCAAGTG	0	0	0	0	0	0	0	0
@SRR111942.39308717-B	chr12	436386	436414	29	TTATTTCCCTTGTGCTTTGATAATTTGAA	KDM5A	KNOWN	protein_coding	0	0	0	0	0
@SRR027545.6070452-A	chr12	1263468	1263485	18	ACACACACACACACATAT	ERC1	KNOWN	protein_coding	0	0	0	0	0
@ERR020157.23436405-B	chr12	5046105	5046127	23	GTTTGTCTGCATTTCCTCTTAAC	0	0	0	0	0	0	0	0
@SRR189827.21496920-A	chr12	9763497	9763515	19	CAGCAGAAGTTTCTACAGC	0	0	0	0	0	0	0	0

@SRR190853.133621692-B	chr12	13660909	13660928	20	AAGACTGTTGTAAAGATTAA	0	0	0	0	0	0	0	0
@SRR015525.2507213-B	chr12	16465025	16465046	22	CTCCCTCTCTCTTTCTGTGTGT	0	0	0	0	0	0	0	0
@ERR018430.18710171-A	chr12	19236691	19236708	18	AAAAAAAAAAACAAAAAA	0	0	0	0	0	0	0	0
@SRR032384.23082759	chr12	26245436	26245463	28	GCTGCAGTAAGTAGCTACAA TAAGTAGC	0	0	0	0	0	0	0	0
@SRR062604.5397634-B	chr12	27371478	27371498	21	GTGTGTGTGTGTGTGTGTACA	0	0	0	0	0	0	0	0
@SRR029893.20027681-B	chr12	29238399	29238431	33	ACAGAATAGTTTCACTGTCCT AAAATTCTGTAC	0	0	0	0	0	0	0	0
@SRR360757.182561951	chr12	32817683	32817700	18	AAAAAAAAAAAAAAAAAA	0	0	0	0	0	0	0	0
@SRR014713.10273315-A	chr12	34845716	34845737	22	ATTGAAGTCACAGAGTTGAC CA	0	0	0	0	0	0	0	0
@ERR020282.36276493-A	chr12	44346933	44346950	18	TCCTGAATGAAGCACTCA	TMEM117	KNOWN	protein_coding	0	0	0	0	0
@SRR029899.19552092	chr12	44856248	44856277	30	TTATTTNTTATTGGTTAAATA GCCAATAAA	0	0	0	0	0	0	0	0
@SRR029934.16842674-A	chr12	45511252	45511269	18	TTTTTTTTTTTTTAAAAA	0	0	0	0	0	0	0	0
@ERR016246.21535219	chr12	48027818	48027835	18	ATGTGTATGTTTACACAT	0	0	0	0	0	0	0	0
@ERR013122.1962936-B	chr12	51719557	51719584	28	TGCAGTGGCACCATCAGGGC TCACTGCA	0	0	0	0	0	0	0	0
@SRR063268.29136048-A	chr12	55922025	55922050	26	GTGTGTGTGTGTGTGTGTGTG TATAT	RP11-110A12 .2	NOVEL	antisense	0	0	0	0	0
@SRR029898.7938051	chr12	57130933	57130957	25	GATCCCTCGCATGCACAGTTC AAAA	PRIM1	KNOWN	protein_coding	0	0	0	0	0
@SRR029859.7672232-B	chr12	61105894	61105918	25	GCACTGACCTTGCCATATGA ACTGC	0	0	0	0	0	0	0	0
@ERR044627.67354458-A	chr12	77766949	77766969	21	ACATAGACAAAAATCCTTTC C	RP1-34H18.1	NOVEL	lincRNA	0	0	0	0	0
@ERR018423.23273133	chr12	78346735	78346761	27	ACAAAACAAGGGAATTTATT GTTTAGT	NAV3	KNOWN	protein_coding	0	0	0	0	0
@ERR009328.6965508-B	chr12	87880254	87880276	23	CTATACACGTGTATAGGCAT ACA	0	0	0	0	0	0	0	0
@ERR020256.5126693	chr12	87902117	87902138	22	ACAAAAAAAAAAAAAAAAAA GGA	0	0	0	0	0	0	0	0
@SRR029894.25493043-B	chr12	105514098	105514133	36	AAAAGTAACTGTGATACAAA AACAGCAATAATTCTT	KIAA1033	NOVEL	protein_coding	0	0	0	0	0
@SRR016203.9082702	chr12	107165065	107165099	35	ATAGCTGGATTGTCCCACAGT GTCCTTAGCTGTTA	RP11-144F15 .1	NOVEL	protein_coding	0	0	0	0	0
@ERR018547.5152124-A	chr12	109782851	109782882	32	TTTCTTGTGTTTTAAGTATAA TGTCTAAGAAA	0	0	0	0	0	0	0	0
@ERR013143.29380372-A	chr12	109881160	109881180	21	TGTGTGTGTGTATGTGTGTGT	MYO1H	KNOWN	protein_coding	0	0	0	0	0
@SRR350098.195287934	chr12	114551303	114551328	26	AATGGAAACTTTCTATTAAA AAACAC	0	0	0	0	0	0	0	0
@SRR017029.3311158-B	chr12	114579093	114579124	32	TAAAGTACATGCCTCACAGG CAGGTCCATTTA	0	0	0	0	0	0	0	0
@ERR013074.9656901-A	chr12	115753414	115753434	21	TCAGTAGACTTACTGAGGAT A	0	0	0	0	0	0	0	0
@SRR189825.40509583-B	chr12	120003634	120003653	20	CATGGTAGCTCGCTCCCATG	RP11-768F21 .1	NOVEL	lincRNA	0	0	0	0	0
@ERR019487.8816370-A	chr12	125116984	125117015	32	TAGCCCCATTTCATGGACGA GGGAATGGGCGA	0	0	0	0	0	0	0	GAT A1,R

														BBP5,UBTF,ZNF263
@SRR065449.6820979-B	chr12	128296324	128296339	16	TCTCTCTCTCTCTCTC	RP11-749H20.1	NOVEL	lincRNA	0	0	0	0	0
@ERR013022.8814078-A	chr12	129560571	129560589	19	CAGCAGCTGTCTCATTTCA	TMEM132D	KNOWN	protein_coding	0	0	0	0	0
@ERR050113.25007686-B	chr12	133010084	133010118	35	AAGATAAGCTTGAACATACAGGCCCATGTAACCTT	0	0	0	0	0	0	0	0
@ERR050114.18057600	chr13	24503386	24503409	24	CACTTACCACTGTGTGGGTAAGTG	0	0	0	0	0	0	0	0
@ERR050125.23535005-B	chr13	51839830	51839859	30	TCCTTAGTGGTTTATGGACTCTGATGATGA	FAM124A	KNOWN	protein_coding	0	0	0	0	0
@SRR111949.14337609	chr13	51977736	51977752	17	TTTTTTTTTTTTCTTTT	INTS6	KNOWN	protein_coding	0	0	0	0	0
@SRR014610.393802	chr13	61798202	61798227	26	ATATCACAGGGGGTTAACACCCCCCC	0	0	0	0	0	0	0	0
@SRR189816.41291770-B	chr13	67411349	67411370	22	CCAAAACGAGAACTCATTTCAG	PCDH9	KNOWN	protein_coding	0	0	0	0	0
@SRR189816.41291770-B	chr13	67411349	67411370	22	CCAAAACGAGAACTCATTTCAG	PCDH9-AS2	KNOWN	antisense	0	0	0	0	0
@ERR018492.74575803	chr13	69418194	69418228	35	TAGAAAATCTCATTAACTTTTCAAAGTAATTTATA	0	0	0	0	0	0	0	0
@SRR032382.21134134	chr13	70903010	70903030	21	TTTTTTAAAAAAAAAAAAAAA	0	0	0	0	0	0	0	0
@SRR032378.18537409	chr13	71240567	71240594	28	TAGTGATATGTAACACTGCTGTTATATA	0	0	0	0	0	0	0	0
@SRR034808.15848819-A	chr13	73346308	73346328	21	CTTTGCCGAGTACTCGAAAGT	DIS3	KNOWN	protein_coding	1	1	0	0	0
@ERR015510.1232695-B	chr13	73773317	73773340	24	CACACACTAGCAACTGTGTGTGTG	0	0	0	0	0	0	0	multiple
@SRR017039.610105	chr13	78556299	78556335	37	AACTGATTTGCATTTCCAACAACAATGCAAACAAGTT	RNF219-AS1	KNOWN	antisense	0	0	0	0	0
@SRR029730.11981834	chr13	99100409	99100428	20	TGGGCATCGCTTACAGAAAC	FARP1	KNOWN	protein_coding	0	0	0	POLR2A	0
@SRR003662.7917012	chr13	99268102	99268119	18	TTTTTTTTTTTTTTTTTT	0	0	0	0	0	0	0	0
@ERR018542.9854541-B	chr14	21656866	21656884	19	CCAACCTGGGAAGCCAGGC	0	0	0	0	0	0	0	0
@ERR042942.44469918	chr14	25133533	25133554	22	GCTGCCCTCGGTAGAGAGCAGC	0	0	0	0	0	0	0	0
@SRR062571.14587782-B	chr14	31562063	31562092	30	AATAGTTTAAAGTTAAAAATTGTTAACAAT	AP4S1	KNOWN	protein_coding	0	0	0	0	0
@SRR360398.1930817-A	chr14	41029064	41029082	19	TATATGTGTGTGTGTGCAT	0	0	0	0	0	0	0	0
@SRR018033.6547876	chr14	41253334	41253356	23	AAGCTAAGTAAACTAAAGAGCTT	0	0	0	0	0	0	0	0
@SRR061639.7634495-A	chr14	46521662	46521692	31	ACTTTTAACTTTTTTCTGTCCTATTATTTCA	LINC00871	NOVEL	lincRNA	0	0	0	0	0
@ERR009225.4077550-A	chr14	51179514	51179530	17	AAAAAAAAAAAAAAAAA	0	0	0	0	0	0	0	0
@SRR029726.2667875	chr14	52642167	52642187	21	ACGACCTCTGGGAGGTAATTA	0	0	0	0	0	0	0	0
@SRR360753.95962803-A	chr14	54655742	54655772	31	ACACACACACACACACACACACACACATATA	0	0	0	0	0	0	0	0

@SRR062615.6548524-B	chr14	55752598	55752630	33	TTAAGGGACTTATGCAAGAG TCCAAAATCTTAA	FBXO34	KNOWN	protein_coding	0	0	0	0	0
@SRR030033.13115369	chr14	59063677	59063706	30	TAAAAGATAGATAGGTAGAT AGATAGTCAG	0	0	0	0	0	0	0	0
@SRR022706.12780321	chr14	62650025	62650052	28	TGGTGCAGGGTAAGCACAGC CTAAACTA	0	0	0	0	0	0	0	0
@SRR061644.20580385-A	chr14	62902298	62902327	30	AATGAGGAGAACAAAGGGTG TTCTACTTAT	0	0	0	0	0	0	0	0
@SRR014710.8299343-B	chr14	85405608	85405624	17	TTTTTTTTTTTTTTTCT	0	0	0	0	0	0	0	0
@SRR062559.16791759-A	chr14	87879618	87879651	34	AGATTGGTTCAACTCAGACA ACCCGGAATAATCT	RP11-594C13 .1	NOVEL	lincRNA	0	0	0	0	0
@SRR026595.20230225-B	chr14	87902499	87902536	38	AAAAAAAAAAAAAACCATGC TTCCACTCCTTAATATTA	RP11-594C13 .1	NOVEL	lincRNA	0	0	0	0	0
@ERR009199.12171395-A	chr14	92941059	92941078	20	TATATACGTGTGTGTGTACA	SLC24A4	KNOWN	protein_coding	0	0	0	0	0
@ERR016289.6531340-B	chr14	95511695	95511721	27	GCACCTCAGGGGACATGTTG TCAGGGT	0	0	0	0	0	0	0	EBF1
@ERR043006.49726549-A	chr14	96049002	96049021	20	ACACACACGTGTGTGCATAT	0	0	0	0	0	0	0	0
@SRR006215.6703383	chr14	98576477	98576494	18	AATTTCTTCATTTTTGAA	RP11-61O1.1	NOVEL	lincRNA	0	0	0	0	0
@SRR350144.852695	chr15	34577898	34577917	20	ACACACACACACACACACAC	SLC12A6	KNOWN	protein_coding	0	0	0	0	0
@SRR043394.14403385-A	chr15	36259778	36259808	31	CACGATTTGACTTCCCCCTTG TTTAAGCGTG	RP11-184D12 .1	NOVEL	lincRNA	0	0	0	0	0
@ERR018555.15797003-B	chr15	36994183	36994215	33	TGAAGTCATAATCCCCTGTTT GTCACATCATAA	C15orf41	KNOWN	protein_coding	1	0	1	0	0
@SRR043212.21943343	chr15	38117643	38117675	33	TAGTATTTTAAACAGACCTTC AGTGGAAAGCTA	0	0	0	0	0	0	0	0
@ERR020241.85419579-B	chr15	40984162	40984188	27	CCAGTTGTACCAGCAGTGTTC ATCTGG	RAD51-AS1	NOVEL	processed_transc ript	0	0	0	0	USF1
@SRR005818.8277212-A	chr15	46254993	46255007	15	AATGAGAACATTAGT	0	0	0	0	0	0	0	0
@SRR100169.108310008-B	chr15	50615189	50615213	25	CACTCCTGTTGCCCAGACAG GAGTG	GABPB1	KNOWN	protein_coding	0	0	0	0	0
@ERR009236.16734012-A	chr15	52283339	52283358	20	GTGTGTGTGTGTGTGTGTGT	MAPK6	KNOWN	protein_coding	0	0	0	0	0
@ERR009236.16734012-A	chr15	52283359	52283377	19	TGTGTGTGTGTGTATATGT	MAPK6	KNOWN	protein_coding	0	0	0	0	0
@ERR018478.79326883	chr15	57514955	57514974	20	AAAAAAAAAAAAAAAAAAA T	TCF12	KNOWN	protein_coding	0	0	0	0	0
@SRR062555.2106638-A	chr15	59508885	59508907	23	GTGTGTGTGTGTGTGTGTATG TG	MYO1E	KNOWN	protein_coding	0	0	0	0	0
@SRR062555.2106638-A	chr15	59508885	59508907	23	GTGTGTGTGTGTGTGTGTATG TG	AC092756.1	NOVEL	miRNA	1	0	0	0	0
@ERR019896.23544444-A	chr15	76195623	76195650	28	TTTAAATAGCATAAGCTCGAT TTATAAA	0	0	0	0	0	0	0	0
@SRR014673.10692139	chr15	78122037	78122061	25	TATAAAAAAAATTTTCTGGAT ACAA	0	0	0	0	0	0	0	0
@ERR006199.16323612-B	chr15	87547520	87547554	35	TTAAACTCTCCCACTATAATT GTGTGTGAGTNTAA	AGBL1	KNOWN	protein_coding	0	0	0	0	0
@ERR023212.3865895-A	chr15	88581438	88581462	25	GGTATACATCAGAAGACATG ATGAA	NTRK3	KNOWN	protein_coding	0	0	0	0	0
@SRR062574.13711024-A	chr15	89373798	89373819	22	ACACAGAGGGAAGTATTTGT GT	ACAN	KNOWN	protein_coding	0	0	0	0	0
@ERR018508.7009988-A	chr15	90814190	90814210	21	GCTGGGATTACAGGCATGAG C	RP11-697E2. 6	NOVEL	protein_coding	0	0	0	0	0

@ERR018508.7009988-A	chr15	90814190	90814210	21	GCTGGGATTACAGGCATGAGC	NGRN	KNOWN	protein_coding	0	0	0	0	0
@SRR350167.139089831-B	chr15	91264858	91264876	19	AACATTTTTTTAAAAGATA	BLM	KNOWN	protein_coding	0	0	0	0	0
@SRR030608.10305473	chr15	94786193	94786211	19	TATGTGTANATACACATCT	MCTP2	KNOWN	protein_coding	0	0	0	0	0
@SRR063271.27655219-A	chr16	3711303	3711324	22	TATGCCCTTTATCTGTTGTAAA	DNASE1	KNOWN	protein_coding	1	0	0	0	0
@SRR063271.27655219-A	chr16	3711303	3711324	22	TATGCCCTTTATCTGTTGTAAA	TRAP1	KNOWN	protein_coding	0	0	0	0	0
@SRR014735.4405997-A	chr16	6547552	6547570	19	TTTTTTTTTAAAAACACAA	RP11-420N3.2	NOVEL	processed_transcript	0	0	0	0	0
@SRR014735.4405997-A	chr16	6547552	6547570	19	TTTTTTTTTAAAAACACAA	RBFOX1	KNOWN	protein_coding	0	0	0	0	0
@SRR043390.2254308	chr16	6726644	6726668	25	GGAAAAAAAAAAGAAAAAAAAAAAA	RP11-420N3.2	NOVEL	processed_transcript	0	0	0	0	0
@SRR043390.2254308	chr16	6726644	6726668	25	GGAAAAAAAAAAGAAAAAAAAAAAA	RBFOX1	KNOWN	protein_coding	0	0	0	0	0
@SRR029762.8060037	chr16	7074015	7074039	25	TAAATCCTTTTTCAACATACGGTTA	RBFOX1	KNOWN	protein_coding	0	0	0	0	0
@SRR062619.1046127	chr16	7646131	7646148	18	TTTCTTTTTTTTTTTTTT	RBFOX1	KNOWN	protein_coding	0	0	0	0	0
@SRR063407.19853527	chr16	7944449	7944467	19	ATACACACACACATACACA	0	0	0	0	0	0	0	0
@ERR018529.5838926-A	chr16	9239856	9239882	27	CACACACACACACACACACACACACAC	0	0	0	0	0	0	0	0
@ERR050123.17547387-A	chr16	9251251	9251277	27	CTGTCCCCTGTGGAAAGCACAGGGGAC	0	0	0	0	0	0	0	0
@SRR062645.20657322-A	chr16	12653895	12653920	26	ATAGTTTTCTAGTCATTTTTCTTTGT	SNX29	KNOWN	protein_coding	0	0	0	0	0
@SRR063272.24026283-B	chr16	13418045	13418063	19	AAAGAAAGGGGGTTTGTTT	U91319.1	NOVEL	lincRNA	0	0	0	0	0
@ERR038236.8296437-A	chr16	33899701	33899718	18	TTCCACTCCACTCCACTC	0	0	0	0	0	0	0	0
@SRR030614.2880420-B	chr16	54313549	54313569	21	TTCACCCACTTCGAGGGTGAA	0	0	0	0	0	0	0	0
@SRR359061.142663327-B	chr16	58342859	58342881	23	ATCTATAAAGAAAGTTATAGATA	GINS3	KNOWN	protein_coding	0	0	0	0	0
@SRR360715.118923450-B	chr16	72265789	72265811	23	TGTCCTCACAAAAGTATATACTT	0	0	0	0	0	0	0	0
@ERR022462.63222001-B	chr16	75067551	75067576	26	AGTCCTCTTGAGGTCAGGAGTTCAAG	ZNRF1	KNOWN	protein_coding	0	0	0	0	CEBPB,NFKB1,RUNX3
@SRR352199.12183645-B	chr16	82164244	82164263	20	ATACACACACACGTGTATAT	RP11-510J16.5	NOVEL	antisense	0	0	0	0	0
@ERR019487.8769861-A	chr17	685605	685626	22	CGCCGCCATGTTCCCTGAGACC	RNMTL1	KNOWN	protein_coding	1	1	1	multiple	0
@SRR032392.7834773	chr17	5840354	5840377	24	ATAGAGCCCAAAGTCTGCGCTCTA	WSCD1	KNOWN	protein_coding	0	0	0	0	0
@ERR020233.6233715-B	chr17	6199553	6199574	22	ACACACACACACACACACACAC	0	0	0	0	0	0	0	0
@SRR016201.7382546	chr17	9330189	9330212	24	ATAGACTGGTAATACTAGGTGTGT	STX8	KNOWN	protein_coding	0	0	0	0	0
@SRR016406.2646730	chr17	10292091	10292116	26	GTTTTCCAGCCTGGGGGACAGAGGCA	RP11-799N11.1	NOVEL	antisense	0	0	0	0	0

@SRR016406.2646730	chr17	10292091	10292116	26	GTTTTCCAGCCTGGGGGACAGAGGCA	CTC-297N7.11	NOVEL	antisense	0	0	0	0	0
@SRR029765.6127463	chr17	12708867	12708886	20	TAAAGACAATACACTCCAAA	ARHGAP44	KNOWN	protein_coding	0	0	0	0	0
@ERR018478.3691567-B	chr17	14294104	14294124	21	ATGCGATAGCCCAAGGGCTGT	AC022816.2	NOVEL	lincRNA	0	0	0	0	0
@SRR015469.13830	chr17	19183456	19183472	17	AGTCACCACTGACAAAG	EPN2	KNOWN	protein_coding	0	0	0	0	0
@SRR075274.1581159-B	chr17	21313436	21313453	18	GGGGACACTGCCAGAGCC	KCNJ12	KNOWN	protein_coding	0	0	0	0	0
@SRR065445.9838520-A	chr17	22134854	22134875	22	TAACATAAGTAATTAAACTATA	0	0	0	0	0	0	0	0
@ERR009263.14142248-A	chr17	25267434	25267452	19	CTGCACTGCACTCCATACT	0	0	0	0	0	0	0	0
@SRR069530.3675779-B	chr17	29120201	29120224	24	TATTATGTAGGGCACTGATATTCC	CRLF3	KNOWN	protein_coding	0	0	0	0	0
@SRR014714.4886534	chr17	38439425	38439442	18	TTTTTTTTGTTTTTTGTT	WIPF2	KNOWN	protein_coding	1	0	1	0	0
@SRR023390.13141367	chr17	41043239	41043256	18	AAAAAAAACCCAAAAAAA	LINC00671	NOVEL	lincRNA	0	0	0	0	0
@ERR015874.6390374-B	chr17	42053360	42053377	18	TATATACACGTACGTGTA	PYY	KNOWN	protein_coding	0	0	0	0	0
@SRR360755.163048407-A	chr17	48381316	48381334	19	AAAAAAAAAGTTTAAAAAA	0	0	0	0	0	0	0	0
@ERR043024.46330867	chr17	55234075	55234105	31	TTTGGGAGGCCGAGGCAGGCGGATCACCTAA	0	0	0	0	0	0	0	0
@ERR005739.8659355-A	chr17	59744589	59744619	31	TAAATTCTATGGTTACATGCAATCACAAAGA	0	0	0	0	0	0	0	0
@ERR022467.94387579-A	chr17	66162444	66162464	21	CAGAGGGAAAAAAAATTTTTT	0	0	0	0	0	0	0	0
@SRR015488.2933468-B	chr17	67238569	67238601	33	AAAAAGAATTCCTCACCTGGGTTGCAATTTTAC	ABCA10	KNOWN	protein_coding	0	0	0	0	0
@ERR012122.18057994-A	chr18	2605299	2605319	21	ACACACACACACACACACACA	NDC80	KNOWN	protein_coding	0	0	0	0	0
@SRR032190.8394860-B	chr18	3926428	3926445	18	TGCTTGTGTGTGTGTATG	DLGAP1	KNOWN	protein_coding	0	0	0	0	0
@SRR015467.12852266	chr18	7127144	7127173	30	CCAACTCCACCTGCACCATGGTGGAGTTGG	0	0	0	0	0	0	0	0
@SRR029845.2178709-B	chr18	7455985	7456010	26	GGCTCTGGAATCTGACTGTCAGAGCC	0	0	0	0	0	0	0	0
@SRR029784.15480134-B	chr18	11427039	11427070	32	AATAAAGGTTGAGATTAAAAATGCCATTGACT	0	0	0	0	0	0	0	0
@SRR062593.991156-A	chr18	12544174	12544191	18	AAAAAAAAAAAAGTTTTT	SPIRE1	KNOWN	protein_coding	0	0	0	0	0
@SRR023315.5409919-A	chr18	18581002	18581027	26	TACAACATATTACTTAGCCATTCATC	ROCK1	KNOWN	protein_coding	0	0	0	0	0
@SRR190850.86995565-B	chr18	37446614	37446634	21	CAAGTTTTGTGCTTAATTTGG	RP11-636O21.1	NOVEL	lincRNA	0	0	0	0	0
@ERR020288.70830335-A	chr18	38471076	38471093	18	GAAAATTATTTTTTTTCT	0	0	0	0	0	0	0	0
@SRR032158.9162670-A	chr18	43717310	43717340	31	AAATGCAATTCAAAGCTGACACAGGGAAACA	0	0	0	0	0	0	0	0
@SRR350142.149307576-A	chr18	47583392	47583412	21	TTTATAGCTCTATAGTATCGT	MYO5B	KNOWN	protein_coding	0	0	0	0	0
@SRR063289.15837474-B	chr18	51028900	51028928	29	TTAGAATCAAAACTTGTTGAAAGCTCTAA	DCC	KNOWN	protein_coding	0	0	0	0	0
@SRR063289.15837474-B	chr18	51028900	51028928	29	TTAGAATCAAAACTTGTTGAAAGCTCTAA	RP11-671P2.1	NOVEL	antisense	0	0	0	0	0
@SRR029826.7253512	chr18	53057563	53057593	31	TAGTATAGTAACCATGAGGC	TCF4	KNOWN	protein_coding	0	0	0	0	0

					CTTAGTTACTA								
@ERR052929.120589285-B	chr18	54692134	54692157	24	ATTCTATAAGGGACATAACT ATCA	WDR7	KNOWN	protein_coding	0	0	0	0	0
@SRR015471.231973	chr18	59839832	59839860	29	TTTGCATTTACATTTACAATT TACAAATA	PIGN	KNOWN	protein_coding	0	0	0	0	0
@SRR005819.4985050-A	chr18	64007014	64007044	31	TAAACACATAATTTGTTAAGT GCTTTTTGTT	0	0	0	0	0	0	0	0
@ERR018421.21468152-B	chr18	67670899	67670915	17	TGGCTCTGTCAGAGCGA	0	0	0	0	0	0	0	0
@ERR018529.3151797-A	chr18	68280985	68281004	20	TACACACACACACACATATA	0	0	0	0	0	0	0	0
@ERR013119.10330551-A	chr18	75892829	75892848	20	TGTGTGTCTCTGTGTGCACA	0	0	0	0	0	0	0	0
@ERR015517.12559596-B	chr19	12387813	12387831	19	TATTTATTTTTTGCAGTTC	ZNF44	KNOWN	protein_coding	0	0	0	0	0
@ERR015517.12559596-B	chr19	12387835	12387857	23	GAACTGGGGTAATCTATAAG AAA	ZNF44	KNOWN	protein_coding	0	0	0	0	0
@SRR075006.59335444-B	chr19	13295945	13295963	19	ACGTATACATACGTGTGTA	0	0	0	0	0	0	0	POL R2A
@SRR062626.18556048-B	chr19	18345921	18345943	23	GGGAGAGGGGGCACTGGTAT GCA	PDE4C	KNOWN	protein_coding	0	0	0	0	0
@SRR111942.27031175-A	chr19	18711667	18711685	19	AGCTATGATCGTGCCACTG	CRLF1	KNOWN	protein_coding	0	0	0	0	0
@ERR019487.6890270-A	chr19	19630102	19630125	24	AGGCCTGAGGTCCTTCTGTGG CCT	NDUFA13	KNOWN	protein_coding	0	0	0	0	0
@ERR019487.6890270-A	chr19	19630102	19630125	24	AGGCCTGAGGTCCTTCTGTGG CCT	YJEFN3	KNOWN	protein_coding	0	0	0	0	0
@ERR019487.6890270-A	chr19	19630102	19630125	24	AGGCCTGAGGTCCTTCTGTGG CCT	CTC-260F20. 3	NOVEL	protein_coding	0	0	0	0	0
@SRR017292.6000842	chr19	19900252	19900270	19	AACAAAAAAAAAAAAAAAA	CTC-559E9.4	NOVEL	lincRNA	0	0	0	0	0
@SRR017292.6000842	chr19	19900252	19900270	19	AACAAAAAAAAAAAAAAAA	CTC-559E9.6	NOVEL	processed_transc ript	0	0	0	0	0
@SRR017292.6000842	chr19	19900252	19900270	19	AACAAAAAAAAAAAAAAAA	ZNF506	KNOWN	protein_coding	0	0	0	0	0
@SRR017292.6000842	chr19	19900252	19900270	19	AACAAAAAAAAAAAAAAAA	CTC-559E9.5	NOVEL	sense_intronic	0	0	0	0	0
@SRR359106.184364257-A	chr19	27732442	27732461	20	GTTCACCTCTGTGAGTTGAA	0	0	0	0	0	0	0	0
@SRR018032.12647333	chr19	27738549	27738566	18	TTCAACTCTGTGAGTTGA	0	0	0	0	0	0	0	0
@ERR018474.24664713-A	chr19	28650133	28650151	19	TATTCACACAGTTTGAAAA	0	0	0	0	0	0	0	0
@SRR029708.853339-B	chr19	33216750	33216768	19	AAATGGAAGAACATTCCAT	TDRD12	KNOWN	protein_coding	0	0	0	0	0
@SRR014787.8561063	chr19	38062908	38062935	28	TAGGACTAATGTACTAATTAT GGCCAGA	ZNF571-AS1	NOVEL	antisense	0	0	0	0	0
@SRR014787.8561063	chr19	38062908	38062935	28	TAGGACTAATGTACTAATTAT GGCCAGA	ZNF540	KNOWN	protein_coding	0	0	0	0	0
@SRR014787.8561063	chr19	38062908	38062935	28	TAGGACTAATGTACTAATTAT GGCCAGA	ZNF571	KNOWN	protein_coding	0	0	0	0	0
@SRR006275.5559844	chr19	40329911	40329941	31	TAGGACAGCCCAGTTCCTTTC AAGGCTCCTA	FBL	KNOWN	protein_coding	0	0	0	0	0
@ERR020255.46931356-A	chr19	46166888	46166905	18	TTGCAGTGAGCCTGGTTC	0	0	0	0	0	0	0	0
@ERR042940.45709259	chr19	48177561	48177580	20	AGGCCTACACAACCTGTGTA	CTD-2571L2 3.8	NOVEL	lincRNA	0	0	0	0	0
@ERR042940.45709259	chr19	48177561	48177580	20	AGGCCTACACAACCTGTGTA	GLTSCR1	KNOWN	protein_coding	0	0	0	0	0

@SRR065443.28113692-A	chr19	50798267	50798289	23	TTTTTTTAAAAAAAAAAAAA ACA	MYH14	KNOWN	protein_coding	0	0	0	0	0
@SRR029832.16846423	chr19	56389841	56389876	36	ATATTAACCAAAAAAAGGTA TGAAGCCGTCTCAGAT	NLRP4	KNOWN	protein_coding	0	0	0	0	0
@ERR042946.44015214	chr19	56505138	56505162	25	CTGTAGCTCTTCATGTGAGCT ACAG	0	0	0	0	0	0	0	0
@SRR062563.29709618-A	chr19	57555171	57555195	25	CCACAATTTATGTGGAGAAG CTACA	0	0	0	0	0	0	0	0
@ERR012613.12190150-A	chr2	2936694	2936715	22	ATGTGCATGTGTGCACATGTG C	AC019118.2	NOVEL	lincRNA	0	0	0	0	0
@ERR020273.54731833-B	chr2	4490936	4490961	26	CAGGTGCTCCTTAGGTACTCT TGTCT	0	0	0	0	0	0	0	0
@SRR030608.10771287	chr2	6603160	6603179	20	TAAAGCAAAATATCAGTTCC	0	0	0	0	0	0	0	0
@SRR029730.2332269	chr2	17907504	17907523	20	AATTTCCTAGATTAGGAATT	SMC6	KNOWN	protein_coding	0	0	0	0	0
@SRR022590.9740177-B	chr2	26894975	26895001	27	AGTCAGGCTTGGGTTTCAATC CTGACT	AC015977.6	NOVEL	antisense	0	0	0	0	0
@SRR190852.94231223-A	chr2	28642959	28642993	35	CTGAAGGCCATCAGACCTGT GTGAGGGCCTGGCAG	0	0	0	0	0	0	0	CTCF,POLR2A
@ERR015759.2384639-B	chr2	30505103	30505131	29	CTTCAGCCGGGACCTGCTGAT GTATAGGG	LBH	KNOWN	protein_coding	0	0	0	0	PAX5,RUNX3,TCF12,TCF3
@SRR061623.12994841-B	chr2	30794355	30794383	29	TAGCTAGTTGTGCAGTAAGC AAAAATCTA	LCLAT1	KNOWN	protein_coding	0	0	0	0	0
@SRR017203.10348682	chr2	31222653	31222683	31	TTTCTTAGGAGATAACTCTTC CATGGAAATA	GALNT14	KNOWN	protein_coding	0	0	0	0	0
@ERR018558.21204241-B	chr2	32595124	32595143	20	TGTGTGTGTGTGTGTATATA	BIRC6	KNOWN	protein_coding	0	0	0	0	0
@ERR015503.9229673-A	chr2	33141336	33141357	22	CCCCCCCCCCCCCCCCCCCCC C	LINC00486	NOVEL	lincRNA	0	0	0	0	multiple
@SRR064387.29748159	chr2	33141373	33141387	15	CCCCCCCCCCCCCCC	LINC00486	NOVEL	lincRNA	0	0	0	0	multiple
@ERR018433.33832850	chr2	33141481	33141500	20	CCCCCCCTCCCCCCCCCCNC	LINC00486	NOVEL	lincRNA	0	0	0	0	0
@ERR022461.35204985	chr2	33141571	33141589	19	CCCCACCCCCCCGCCCCCC	LINC00486	NOVEL	lincRNA	0	0	0	0	multiple
@SRR029679.5324047-B	chr2	33141627	33141648	22	CGGCCCCCCCCCGCCCCCCC GC	LINC00486	NOVEL	lincRNA	0	0	0	0	multiple
@SRR189829.72222224	chr2	37503930	37503948	19	TATATGTGTGTGTGTGTGT	PRKD3	KNOWN	protein_coding	0	0	0	0	0
@ERR013022.15704088-A	chr2	44093022	44093052	31	TTCTATCCAGAGAGTGAGGA TTCTATCAAGA	ABCG8	KNOWN	protein_coding	0	0	0	0	0
@ERR018528.9603621-B	chr2	44183635	44183652	18	ACACATACACACACACAC	LRPPRC	KNOWN	protein_coding	0	0	0	0	0
@ERR009257.10352326-A	chr2	44545742	44545769	28	TATGTTTGCATAGGCACAGA ATGTATCA	SLC3A1	KNOWN	protein_coding	1	0	1	0	0
@ERR009257.10352326-A	chr2	44545742	44545769	28	TATGTTTGCATAGGCACAGA ATGTATCA	PREPL	KNOWN	protein_coding	1	0	1	0	0
@ERR009382.10202676-A	chr2	48192379	48192407	29	CTGATTGGCTATGGGAGGGG GGCAATTAG	AC079807.4	NOVEL	lincRNA	0	0	0	0	NFYB

@ERR013168.20544084	chr2	52906939	52906955	17	GTGTGTGTGTTTGTGTG	0	0	0	0	0	0	0	0
@ERR009330.3843309-A	chr2	54310226	54310245	20	AAAAATAGTCACTATTTTCT	ACYP2	KNOWN	protein_coding	0	0	0	0	0
@ERR009330.3843309-A	chr2	54310226	54310245	20	AAAAATAGTCACTATTTTCT	RP11-477N3.1	NOVEL	lincRNA	0	0	0	0	0
@SRR038710.20282927-B	chr2	60691734	60691754	21	CTTCGATTTCAATCGAGGTCT	BCL11A	KNOWN	protein_coding	0	0	0	0	0
@ERR020237.69766813-A	chr2	63358254	63358269	16	TGGGTTTTTTTAAAAA	WDPCP	KNOWN	protein_coding	0	0	0	0	0
@ERR022429.24536962	chr2	64117420	64117448	29	TAATTACACTAGTAATTAAGGTGGCACTA	UGP2	KNOWN	protein_coding	0	0	0	0	0
@ERR019905.3694311-A	chr2	65832114	65832134	21	CTCTCTCACACACACACACAC	AC074391.1	NOVEL	lincRNA	0	0	0	0	0
@ERR019905.3694311-A	chr2	65832114	65832134	21	CTCTCTCACACACACACACAC	AC007389.3	NOVEL	lincRNA	0	0	0	0	0
@SRR061648.5850060-A	chr2	68903113	68903135	23	TTTTTTTTAAAAAAAAAAAAAAA	0	0	0	0	0	0	0	0
@ERR016275.23485569-B	chr2	77022341	77022382	42	AAAAATGATTTTTAAAGTACAAAACACACTTTTCATTAATTT	LRRTM4	KNOWN	protein_coding	0	0	0	0	0
@SRR017033.11291613-A	chr2	77265463	77265487	25	TTAACATGCCTAAGGTCAAGTGAAT	LRRTM4	KNOWN	protein_coding	0	0	0	0	0
@ERR018423.20326972-B	chr2	78347141	78347165	25	TTTATATTTCTCTCTAAATATAGCA	AC012494.1	NOVEL	lincRNA	0	0	0	0	0
@SRR029726.12410981	chr2	80741977	80742001	25	GTTCTTTCTGAATAATGTCTTTTTA	CTNNA2	KNOWN	protein_coding	0	0	0	0	0
@SRR014131.1972303	chr2	81854668	81854700	33	TAGATTAGTATGGCTTTAGTTAATAACAATCTA	0	0	0	0	0	0	0	0
@ERR050094.1960183	chr2	89130146	89130174	29	TTCCAATTTTTGCTAAAATTTGAAAATCT	AC096579.13	NOVEL	processed_transcript	0	0	0	0	0
@SRR062584.17992766-A	chr2	89850114	89850135	22	TCGAATGGAATGGACTCGAATG	0	0	0	0	0	0	0	0
@ERR009233.12813488-B	chr2	89865480	89865500	21	TCGAATGGAATGGAAAGGAAT	0	0	0	0	0	0	0	0
@SRR032380.22109784	chr2	89867489	89867509	21	TTCACTGGAATGGAATGGGTT	0	0	0	0	0	0	0	0
@ERR019493.11118400-B	chr2	96211321	96211341	21	GTGTATACACTCTGTTATAGA	0	0	0	0	0	0	0	0
@SRR064187.56024561	chr2	97917057	97917079	23	AGATAGAATGAAAAAGAATTATA	ANKRD36	KNOWN	protein_coding	1	0	1	0	0
@ERR044609.68370074-B	chr2	101635025	101635055	31	TCCACATTAAAGGATGTCCTTAAAATGTGGA	RPL31	KNOWN	protein_coding	0	0	0	0	0
@ERR044609.68370074-B	chr2	101635025	101635055	31	TCCACATTAAAGGATGTCCTTAAAATGTGGA	TBC1D8	KNOWN	protein_coding	0	0	0	0	0
@SRR190853.115581746-B	chr2	105655171	105655192	22	TGCATCTTTCCATAGAGGGAGA	MRPS9	KNOWN	protein_coding	0	0	0	ZNF263	0
@SRR350165.115364266-B	chr2	107631369	107631390	22	TATGTGTGTGTGTGTGTATGTG	0	0	0	0	0	0	0	0
@ERR019907.22779831-B	chr2	118095283	118095302	20	ATACTGCTAAGCAATGTAAT	0	0	0	0	0	0	0	0
@SRR063411.18273310	chr2	126373405	126373426	22	TATATGTGTGTGTGTACACACA	0	0	0	0	0	0	0	0
@SRR014764.935911-A	chr2	134937079	134937110	32	GTTTGGTCTTGTTCTATAAAGTCTCTGCATTC	MGAT5	KNOWN	protein_coding	0	0	0	0	0
@SRR058959.26200638	chr2	140991668	140991700	33	TTATAATGTTTCCTTGTTGTAACTAAGATTTAA	LRP1B	KNOWN	protein_coding	0	0	0	0	0

@SRR063261.7182225-A	chr2	141481114	141481131	18	TTTGCTAACATAATTTAA	LRP1B	KNOWN	protein_coding	0	0	0	0	0
@SRR061665.24983092-B	chr2	142334878	142334898	21	TAGGGGTGTAGGTCACCCCTA	LRP1B	KNOWN	protein_coding	0	0	0	0	0
@SRR350098.175009123	chr2	148370841	148370869	29	AAGATCAGTCAAGCTGTGGTTCTAGACAT	0	0	0	0	0	0	0	0
@ERR018542.5109238-A	chr2	150647106	150647123	18	AAAAAAAAAAACAAAAAG	AC144449.1	NOVEL	antisense	0	0	0	0	0
@ERR018542.5109238-A	chr2	150647106	150647123	18	AAAAAAAAAAACAAAAAG	AC007364.1	NOVEL	lincRNA	0	0	0	0	0
@SRR360581.24389966	chr2	152607227	152607248	22	TCTCTCTCTCTCTCTCGATATA	0	0	0	0	0	0	0	0
@SRR062573.7260628-A	chr2	155002676	155002693	18	GTGTGTGTGTGTATGTAT	GALNT13	KNOWN	protein_coding	0	0	0	0	0
@SRR062573.7260628-A	chr2	155002676	155002693	18	GTGTGTGTGTGTATGTAT	AC008166.1	NOVEL	miRNA	1	0	0	0	0
@ERR018555.9904810-A	chr2	161395431	161395461	31	TATGGGCATGGGCACAAAGTATGAAGATCTA	0	0	0	0	0	0	0	FOXA1
@ERR020275.6034377-B	chr2	165043041	165043060	20	GTTACTTATTTGACAATAAG	AC092684.1	NOVEL	lincRNA	0	0	0	0	0
@SRR018035.8989837	chr2	166356930	166356949	20	GTGTGTGTGTATGTGTGTGT	CSRNP3	KNOWN	protein_coding	0	0	0	0	0
@SRR075009.35241925-B	chr2	176125302	176125320	19	TGTTTATTTGTGTGTATGT	AC096649.3	NOVEL	lincRNA	0	0	0	0	0
@ERR043006.32829743-B	chr2	180262389	180262405	17	ACGTATATGTGTGTGTA	0	0	0	0	0	0	0	0
@SRR015488.1743947	chr2	183201687	183201709	23	TTTTTTTAAAAAAAAAAAATAAA	PDE1A	KNOWN	protein_coding	0	0	0	0	0
@ERR018521.96177-A	chr2	192564195	192564213	19	CGTGCACGTGTGTATACAT	0	0	0	0	0	0	0	POLR2A
@SRR061653.27485240-A	chr2	193547462	193547481	20	TATACGTGTGTATACGTATA	0	0	0	0	0	0	0	0
@ERR018547.12427864-A	chr2	194357217	194357237	21	AGACTGTTGCTTCATCTGTTT	0	0	0	0	0	0	0	0
@ERR044604.52461810-A	chr2	196195945	196195972	28	CTCCCAAGCTGGCCACTCACCTTGGGAG	0	0	0	0	0	0	0	0
@ERR022471.7732164-B	chr2	211152344	211152366	23	AGAGGAAGCCACTCCAGCCCTCT	0	0	0	0	0	0	0	0
@SRR424293.29394159-A	chr2	211182748	211182766	19	TTTTTTTTTTTGTTTTTAT	0	0	0	0	0	0	0	0
@SRR062610.14227667-B	chr2	211622048	211622082	35	AAAAATAAACTGAAAAACAGTATTTCTCAATTTTT	0	0	0	0	0	0	0	0
@SRR006188.10734802-A	chr2	212236019	212236047	29	CTCCTCATGAGTAAAAGAATCATGAGGAG	0	0	0	0	0	0	0	0
@ERR018480.14413477-A	chr2	221131067	221131097	31	ACTTTAAGAAATATCAGTCCCACTATAAGGT	AC114765.1	NOVEL	lincRNA	0	0	0	0	0
@SRR360541.20703462-B	chr2	230983931	230983949	19	TATAACTTAATAAGTTATA	0	0	0	0	0	0	0	0
@SRR385767.34969587-A	chr20	12961227	12961241	15	GTGTGTGTGTGTGTG	0	0	0	0	0	0	0	0
@SRR385767.34969587-A	chr20	12961261	12961275	15	CTCTCTCTCTCTCTC	0	0	0	0	0	0	0	0
@SRR385767.34969587-A	chr20	12961287	12961302	16	TCTCTCTCTCTCTGTG	0	0	0	0	0	0	0	0
@ERR042968.29031609-A	chr20	15001646	15001667	22	TATGCATACACACACGTGTGTG	MACROD2	KNOWN	protein_coding	0	0	0	0	0
@SRR063105.22833660-B	chr20	22724459	22724480	22	TGTGTGTGTGTGTGTGTGTAGG	0	0	0	0	0	0	0	0
@ERR018545.16929984	chr20	25538817	25538850	34	AGGCAGTAAATAAATGAAGACTTAATTACTGCCT	NINL	KNOWN	protein_coding	0	0	0	0	0
@SRR006143.9677653-B	chr20	26288272	26288290	19	CAGCTCGGAGAGTTGAACA	0	0	0	0	0	0	0	0

@SRR023392.10669965	chr20	33263847	33263865	19	AATAAAAAACAAAAAACAA	PIGU	KNOWN	protein_coding	0	0	0	0	0
@SRR189830.61295478	chr20	35238190	35238209	20	ATCAGTGGGGTCCCCTCCTG	TGIF2-C20orf24	KNOWN	protein_coding	0	0	0	0	0
@SRR189830.61295478	chr20	35238190	35238209	20	ATCAGTGGGGTCCCCTCCTG	C20orf24	KNOWN	protein_coding	0	0	0	0	0
@ERR018540.17394802-B	chr20	35607805	35607822	18	GAAATACCTTCAGTATTT	0	0	0	0	0	0	0	0
@ERR052839.34283143-B	chr20	39955092	39955112	21	GTATAAGAGCTTCCCTTATAC	0	0	0	0	0	0	0	0
@ERR044606.15366547	chr20	49325722	49325747	26	GATTGGAATGAGTAATACCAAACAGA	0	0	0	0	0	0	0	ESR1,FOXA1,GATA3
@SRR350098.169145099	chr20	49706498	49706526	29	TTGTACATTTAATTTAAAATGCACAATAA	0	0	0	0	0	0	0	0
@ERR016343.23541943-B	chr20	50217737	50217770	34	CTACTCAAAGCTCACATCATAGGCCGTGCGTTCG	ATP9A	KNOWN	protein_coding	1	1	1	0	MAFK
@SRR016595.7645928	chr20	51630503	51630526	24	TATCATCCCCACGAGGCAGGAAAT	TSHZ2	KNOWN	protein_coding	0	0	0	0	0
@SRR022669.8889733-B	chr20	52629619	52629636	18	TGTATGGTTGATGGTAAC	BCAS1	KNOWN	protein_coding	0	0	0	0	0
@SRR029761.8952329-B	chr20	58421713	58421734	22	AATTTTAGGGTCTTTATACCAC	PHACTR3	KNOWN	protein_coding	0	0	0	0	0
@ERR018545.16998060	chr20	62238765	62238784	20	AGGGGCATGTTTCTAGAAAT	GMEB2	KNOWN	protein_coding	0	0	0	0	0
@ERR018545.16998060	chr20	62238785	62238800	16	AAATGGAGGTGAGGTC	GMEB2	KNOWN	protein_coding	0	0	0	0	0
@SRR047737.239394-B	chr20	62751728	62751748	21	CGCGCACACACACACGCGCAC	0	0	0	0	0	0	0	multiple
@SRR061650.5567546-B	chr21	9860978	9860998	21	ATATAGAGAGAGAGAGAGAGA	0	0	0	0	0	0	0	0
@SRR023875.7188593	chr21	11057647	11057675	29	AATCTCTGCTAGCTCCTTTTCATGATTCA	BAGE2	KNOWN	pseudogene	0	0	0	GATA3, TCF7L2	0
@ERR020258.41137737-B	chr21	26842133	26842151	19	TAATTCCTAAAAAAATAAA	0	0	0	0	0	0	0	0
@ERR012617.13391406-B	chr21	29870006	29870030	25	TATACATACACACACTAACAATTAG	AF131217.1	NOVEL	lincRNA	0	0	0	0	0
@SRR011067.1436781-A	chr21	31405336	31405361	26	CCCTGACCTACAGTGAGTGGTCAGGG	0	0	0	0	0	0	0	0
@SRR014771.4470514-B	chr21	33885374	33885405	32	TAGTCACAAATATCTTACAATAATAACAACTA	EVA1C	KNOWN	protein_coding	0	0	0	0	0
@SRR029677.22996103-B	chr21	39800462	39800480	19	CCGTCCTACTTTAGGACCG	ERG	KNOWN	protein_coding	0	0	0	0	0
@SRR360608.90098719-B	chr22	17205216	17205237	22	GTGTGTGTGTGTGTGTGTGTTT	0	0	0	0	0	0	0	0
@ERR015522.10844057-B	chr22	19347285	19347303	19	GGGAGACTTTGGGGTCTTT	HIRA	KNOWN	protein_coding	0	0	0	0	0
@ERR015522.10844057-B	chr22	19347285	19347303	19	GGGAGACTTTGGGGTCTTT	C22orf39	KNOWN	protein_coding	0	0	0	0	0
@ERR018540.2348203	chr22	22279685	22279708	24	GCTCCTACAAATCTCATAGAAACC	PPM1F	KNOWN	protein_coding	1	0	1	0	0
@SRR026648.1329241-B	chr22	24497924	24497951	28	CAGTTATGAAGATGGAGACATGCTACTA	CABIN1	KNOWN	protein_coding	0	0	0	CTCF	0
@SRR026648.1329241-B	chr22	24497924	24497951	28	CAGTTATGAAGATGGAGACATGCTACTA	KB-318B8.7	NOVEL	sense_intronic	0	0	0	CTCF	0
@SRR014626.7045905-B	chr22	26951219	26951244	26	TCTGGTGTTACTTGCAATGGCAAAAA	TPST2	KNOWN	protein_coding	0	0	0	0	0

@SRR014626.7045905-B	chr22	26951219	26951244	26	TCTGGTGTTACTTGCAATGGCAAAAA	MIR548J	KNOWN	miRNA	1	0	0	0	0
@ERR044623.99325376-A	chr22	27119065	27119085	21	ATGTTGTTAACATTTAGTGTC	CTA-211A9.5	NOVEL	lincRNA	0	0	0	0	0
@SRR359097.169573491-A	chr22	28767571	28767591	21	AAAAAAAATTTTTTTTTTTTT	TTC28	KNOWN	protein_coding	0	0	0	0	0
@ERR022470.67194184-A	chr22	31291521	31291547	27	TACAAAATTAGAAAGTAGAATTTTCTA	OSBP2	KNOWN	protein_coding	0	0	0	0	0
@SRR063304.16446885-B	chr22	32382378	32382400	23	GTCATACACTACCTAGTAATATA	0	0	0	0	0	0	0	0
@SRR063304.16446885-B	chr22	32382406	32382426	21	ACACACACACACATACACATA	0	0	0	0	0	0	0	0
@SRR017038.15013271-B	chr22	32636691	32636709	19	ACCCGCCTGACAGGTAGGT	RP1-90G24.10	NOVEL	antisense	0	0	0	0	CEBPB,GATA3
@SRR017038.15013271-B	chr22	32636691	32636709	19	ACCCGCCTGACAGGTAGGT	SLC5A4	KNOWN	protein_coding	0	0	0	0	CEBPB,GATA3
@ERR018538.7684398-A	chr22	41137054	41137075	22	TCCCATTCATGAGGGTACTGCC	0	0	0	0	0	0	0	TCF7L2
@SRR064182.64246537	chr22	43385893	43385910	18	ATATGTGTGTGTGTATAT	PACSIN2	KNOWN	protein_coding	0	0	0	0	0
@ERR018466.24070753-A	chr3	1506059	1506090	32	GAAATAATGACTTCTCAGATTCAAAGAGAATC	0	0	0	0	0	0	0	0
@SRR360716.120092918-B	chr3	7106202	7106220	19	TGTGTGTGTGTGTGTGTGT	GRM7	KNOWN	protein_coding	0	0	0	0	0
@SRR350165.126501756-A	chr3	7407355	7407377	23	TATATGTGTGTGTGTGTATGTAT	GRM7	KNOWN	protein_coding	0	0	0	0	0
@ERR020283.32741020-A	chr3	21586043	21586064	22	TTGTCAAAATTAATTTGCAGAA	ZNF385D	KNOWN	protein_coding	0	0	0	0	0
@ERR020283.32741020-A	chr3	21586043	21586064	22	TTGTCAAAATTAATTTGCAGAA	ZNF385D-AS1	NOVEL	antisense	0	0	0	0	0
@SRR063405.23659712	chr3	22005925	22005943	19	TTTTTCAATTTTTTTTTTT	ZNF385D	KNOWN	protein_coding	0	0	0	0	0
@SRR063405.23659712	chr3	22005925	22005943	19	TTTTTCAATTTTTTTTTTT	ZNF385D-AS2	NOVEL	lincRNA	0	0	0	0	0
@SRR360717.163431182-A	chr3	24217370	24217399	30	CTAACCTTTTCCCTTTGTAATTATGTACAG	THRB	KNOWN	protein_coding	0	0	0	0	0
@SRR014688.220896-A	chr3	24291024	24291054	31	TGTTTAAGTTCTGGGTTAGAGGTAATCAATC	THRB	KNOWN	protein_coding	0	0	0	0	0
@ERR050101.13008465-B	chr3	26785001	26785019	19	GAGAACTCTGTATGTTCTC	0	0	0	0	0	0	0	0
@SRR360545.27222795-B	chr3	31903976	31904000	25	TTTGTTTTTAGAAAATAAAAAAAAA	OSBPL10	KNOWN	protein_coding	0	0	0	0	0
@SRR029898.976743	chr3	34219952	34219979	28	AATAGTAATCTCTTGAAATTTCTTTTAA	AC018359.1	NOVEL	lincRNA	0	0	0	0	0
@ERR009205.16327670-A	chr3	34708109	34708143	35	TAGTACTACTCTGGCCCATTGGTTTATTGTTTCTA	0	0	0	0	0	0	0	0
@SRR360555.99017356-A	chr3	35077726	35077743	18	CTCTCTCTCTCTCTCTCT	0	0	0	0	0	0	0	0
@ERR019907.20799945-B	chr3	42518211	42518242	32	TTTTGTTTCATTTCTGGTGGGGGGTGCGGAAA	0	0	0	0	0	0	0	0
@ERR018528.9776346-A	chr3	48460455	48460477	23	CTGCCCAACAGCTGTGTTCTCTG	PLXNB1	KNOWN	protein_coding	0	0	0	0	0
@SRR359064.91752006-A	chr3	50967556	50967584	29	AAGATCTATAAAAAACTTAG	DOCK3	KNOWN	protein_coding	0	0	0	0	0

					AGTGTTCCT								
@SRR062565.3605974-A	chr3	59754337	59754356	20	TGACATTGATGTCTGGGAAG	FHIT	KNOWN	protein_coding	0	0	0	0	0
@SRR031785.13707707-B	chr3	60103239	60103268	30	TTACCTAGGTGAAAAACAGT GTCTTTTTTT	FHIT	KNOWN	protein_coding	0	0	0	0	0
@ERR013137.4167691-B	chr3	60116511	60116529	19	TGTGTGTGTATATACACAC	FHIT	KNOWN	protein_coding	0	0	0	0	0
@SRR023376.11257442-B	chr3	61034894	61034919	26	TATTTGGTAAAATTGGTTAAC AACTA	FHIT	KNOWN	protein_coding	0	0	0	0	0
@ERR009277.9559692-B	chr3	65142329	65142353	25	CATGGCAGCACCCTCATGTCC CATG	0	0	0	0	0	0	0	0
@SRR015529.13506325-A	chr3	65404900	65404921	22	TTGTGTGTGTGTGTGTATGTG T	MAGI1	KNOWN	protein_coding	0	0	0	0	0
@SRR189815.35716610-B	chr3	68264030	68264048	19	CCCCACTCATTCAGTGGTG	FAM19A1	KNOWN	protein_coding	0	0	0	0	0
@ERR013094.15636853-A	chr3	71593172	71593191	20	TGTGTGTGTATATGTGTGTA	FOXP1	KNOWN	protein_coding	0	0	0	RUNX3	0
@SRR029928.2675232-A	chr3	75949301	75949318	18	GTGTGTGTGTGTGTGTGT	0	0	0	0	0	0	0	0
@ERR050165.13646622-B	chr3	84879371	84879401	31	AATTGTTAACAATAATTGTTT TTACCATAAT	LINC00971	NOVEL	lincRNA	0	0	0	0	0
@ERR016162.7364381-B	chr3	85078084	85078113	30	CATACTGTTTTATTTCGGTAA AACAGTATG	CADM2	KNOWN	protein_coding	0	0	0	0	0
@ERR018544.13123463-B	chr3	97937233	97937251	19	GCAGCTCAATATACAGAGA	0	0	0	0	0	0	0	0
@ERR022470.40920988	chr3	109535914	109535929	16	GCTTTTTTTTTTTTTT	0	0	0	0	0	0	0	0
@ERR018443.8207967-A	chr3	113528934	113528969	36	CTGCAGTGAGCCAAGATCAC GCCATTGCACTCCAGC	ATP6V1A	KNOWN	protein_coding	1	0	1	0	0
@SRR100169.44114552-B	chr3	116148581	116148606	26	CAAGGCCTGTGTTATCCCAG GCCTTG	LSAMP	KNOWN	protein_coding	0	0	0	0	0
@ERR020282.96048630-B	chr3	119415594	119415622	29	AGCTAAATGTAAGTAAACAT GGATTTAAA	0	0	0	0	0	0	0	0
@ERR018542.16067927-B	chr3	127555515	127555555	41	GTGAGAGGGAGTCCTGTCGG CCTTCTCAGGCCCTGGGCCAG	0	0	0	0	0	0	0	multi ple
@ERR019900.9311843	chr3	138507824	138507842	19	TTTTTTTGGTGTTTTGTTT	PIK3CB	KNOWN	protein_coding	0	0	0	0	0
@SRR014703.2856468	chr3	145566783	145566812	30	AATTATTACCTCTCTCTTTCTT TTAAATAT	0	0	0	0	0	0	0	0
@ERR044612.13690165-B	chr3	153000803	153000820	18	TATAGTTAATGTGTTATA	0	0	0	0	0	0	0	0
@SRR032394.14121258	chr3	158708661	158708676	16	GATACCCAGGCAATCA	IQCJ-SCHIP1	KNOWN	protein_coding	0	0	0	0	0
@SRR032394.14121258	chr3	158708661	158708676	16	GATACCCAGGCAATCA	IQCJ	KNOWN	protein_coding	0	0	0	0	0
@ERR016335.4310078-B	chr3	162750699	162750715	17	ACCTATTTGGTCCTTAA	RP11-10O22. 1	NOVEL	lincRNA	0	0	0	0	0
@ERR022452.82732649-A	chr3	170821848	170821879	32	TAATTGAAAATTTCCTTTAAG TTTAATTGTTA	TNIK	KNOWN	protein_coding	0	0	0	0	0
@ERR020280.44605669-B	chr3	173764850	173764868	19	TACGGCAGAGAAAGATGGA	NLGN1	KNOWN	protein_coding	0	0	0	0	0
@SRR017210.5564190	chr3	176478613	176478632	20	TCTATTTACTCAAAGAATAT	RP11-644C3. 1	NOVEL	lincRNA	0	0	0	0	0
@ERR044611.30080838-B	chr3	182690553	182690582	30	GTGCTGGGATTACAGGTGTG AGCCACTGCG	DCUN1D1	KNOWN	protein_coding	0	0	0	0	0
@SRR352222.30759089-A	chr3	183251922	183251936	15	AAAGTACTTTAAAAG	KLHL6	KNOWN	protein_coding	0	0	0	0	multi ple
@ERR016261.32985316-A	chr3	190866779	190866797	19	CATACCAGAAGCTCTGGGA	0	0	0	0	0	0	0	0

@SRR029722.18472049	chr3	195111920	195111952	33	GAGAAGCTTTTTTTGTATTTT TTAACAAAAAAA	ACAP2	KNOWN	protein_coding	0	0	0	0	0
@SRR100169.58560736-B	chr4	7947622	7947650	29	TTATTGATAACTGTGGTTATT GATTAGTT	0	0	0	0	0	0	0	0
@ERR016343.7180064-B	chr4	8003005	8003029	25	CTATGTGGCACCCCGTGTCCC TCTG	ABLIM2	KNOWN	protein_coding	0	0	0	0	0
@SRR063411.20030177	chr4	8717574	8717610	37	AATGATTTAAAAACAGATCA CTGATTTTAAACCATTT	0	0	0	0	0	0	0	0
@SRR062606.8914113-A	chr4	10176827	10176853	27	GCACAGACGCACCTCTGGGT CTATAGT	0	0	0	0	0	0	0	0
@ERR012606.10323803-B	chr4	10208651	10208677	27	TGTAATCCCATAAGAGACAA GGCAACA	0	0	0	0	0	0	0	0
@SRR189825.69248183-B	chr4	10700909	10700925	17	TGTGTGTGTATACACAC	0	0	0	0	0	0	0	0
@ERR016158.17294294-B	chr4	22589893	22589914	22	AATAATGCTCTTAGTTATTAT T	0	0	0	0	0	0	0	0
@SRR044231.16966648-A	chr4	23759945	23759975	31	GTTACTTTTATTGTTGTTGTTT CATGATCTA	RP11-380P13 .1	NOVEL	antisense	0	0	0	0	0
@SRR044231.16966648-A	chr4	23759945	23759975	31	GTTACTTTTATTGTTGTTGTTT CATGATCTA	PPARGC1A	KNOWN	protein_coding	0	0	0	0	0
@SRR063350.14256320-B	chr4	31472309	31472339	31	TGCATGTTCTACCTTATAAAT GGGAACTATA	0	0	0	0	0	0	0	0
@ERR016158.25358756	chr4	34908895	34908920	26	TTGCTTTCTTCAAGTAACTTT GAAGA	0	0	0	0	0	0	0	0
@ERR016326.20212400-B	chr4	49157329	49157353	25	TCAACCAGACTGGAGTGCAG TGGCA	0	0	0	0	0	0	0	0
@SRR062595.19992148-A	chr4	49644548	49644569	22	TCCCATTCCATTCCTTTCCAA T	0	0	0	0	0	0	0	0
@SRR015523.13543882-A	chr4	54752564	54752583	20	ATTTTGTAGCACTGGATTTG	FIP1L1	KNOWN	protein_coding	0	0	0	0	0
@SRR075010.47523988-A	chr4	56971193	56971210	18	TTCTTTTTTAATTTTTTA	0	0	0	0	0	0	0	0
@ERR015516.9666816-A	chr4	62022429	62022450	22	TTTTTTTTTTTTTTTTTTTTTT	0	0	0	0	0	0	0	JUN D
@SRR061644.9083475	chr4	64431892	64431915	24	TACGTGGTTAGCTAAAACAT GGAT	0	0	0	0	0	0	0	0
@ERR050159.23135523-A	chr4	74166069	74166091	23	TATGTAAACACACACACATA CAC	RP11-692D12 .1	NOVEL	antisense	0	0	0	0	0
@ERR019497.1959212-B	chr4	77681025	77681059	35	TAGCTTACCTGCCCTGCCTAC TCACAGAAAAGCTA	SHROOM3	KNOWN	protein_coding	0	0	0	0	0
@ERR019497.1959212-B	chr4	77681025	77681059	35	TAGCTTACCTGCCCTGCCTAC TCACAGAAAAGCTA	RP11-359D14 .3	NOVEL	antisense	0	0	0	0	0
@SRR075273.104177861	chr4	80201400	80201424	25	GAACTGTGCCTCAAAAAGAG GCACA	LINC01088	NOVEL	antisense	0	0	0	0	0
@SRR075273.104177861	chr4	80201400	80201424	25	GAACTGTGCCTCAAAAAGAG GCACA	NAA11	KNOWN	protein_coding	0	0	0	0	0
@SRR065219.17533524	chr4	80651772	80651790	19	TGTGTGTGTGTATGTATGT	0	0	0	0	0	0	0	0
@SRR064182.73737361	chr4	83655870	83655885	16	TTTTTTAAAAAAAAAA	SCD5	KNOWN	protein_coding	0	0	0	0	0
@SRR015498.2813800-A	chr4	86528899	86528926	28	TAAATGAGGAAGTATTTTTG ATACGGCC	ARHGAP24	KNOWN	protein_coding	0	0	0	0	multi ple
@ERR044612.32847807-A	chr4	91500165	91500186	22	AATTGAGTCAAGCTTTTTTTA A	CCSER1	KNOWN	protein_coding	0	0	0	0	0
@SRR018032.12392293	chr4	92514185	92514218	34	TAGAATTTATGGGGGAGGGG AGACATACTTCAAT	CCSER1	KNOWN	protein_coding	0	0	0	0	0

@SRR015989.4801085	chr4	100032665	100032694	30	GAAGGAAGAGTGTGAGTTAACTCTTCCTTC	RP11-696N14.1	NOVEL	antisense	0	0	0	0	0
@SRR031338.19152516	chr4	107759423	107759445	23	TCTGACACTCTGTGAGAGTCTGA	0	0	0	0	0	0	0	0
@SRR350153.79814386-A	chr4	118662365	118662382	18	AATTTTTTTAAAATTTTT	0	0	0	0	0	0	0	0
@SRR061672.15117694-A	chr4	125748711	125748734	24	CCTGCCATAAATGAAGCTTGCAAG	0	0	0	0	0	0	0	0
@SRR032378.14498144	chr4	125896260	125896285	26	TAGGCTGAAGGCTATAACTTTGATGA	0	0	0	0	0	0	0	0
@SRR029675.22576114	chr4	126781673	126781699	27	AATGTAATTGAAAAGACGTAAACTATA	0	0	0	0	0	0	0	0
@ERR013119.14864810	chr4	126845021	126845039	19	TATGTGTGTGTGTATGTGT	0	0	0	0	0	0	0	0
@SRR043380.8408648-A	chr4	127770556	127770580	25	TGCTTTTTTTTTTTTAAAAAAAGTA	0	0	0	0	0	0	0	0
@SRR043207.29881143	chr4	150375543	150375560	18	GCGCTCACTTACAAGTGG	RP11-526A4.1	NOVEL	lincRNA	0	0	0	0	0
@SRR030031.7444098	chr4	151916687	151916718	32	AATCAAATGTTTCTATCCAGAAACATTACAAT	LRBA	KNOWN	protein_coding	0	0	0	0	0
@ERR018431.13430341-B	chr4	157161798	157161835	38	TTTCAGGATATGCAGTCAACGTGCGATCTGGGAGAAAA	0	0	0	0	0	0	0	0
@SRR359847.1095580-A	chr4	160947126	160947144	19	TCTGTGTGTGTGTGTATAT	0	0	0	0	0	0	0	0
@SRR022706.13454050-A	chr4	163276307	163276324	18	TAGCCCTCTATGTGTCTA	0	0	0	0	0	0	0	0
@SRR014742.11271142	chr4	165282173	165282196	24	ACCCTAGACCATCAAGTGCTCTAG	1-Mar	KNOWN	protein_coding	0	0	0	0	0
@SRR043219.17154216-A	chr4	166457665	166457688	24	GCTAGACGTTTGTCTAGCTTTGTT	0	0	0	0	0	0	0	0
@SRR032158.13928872-A	chr4	178457775	178457792	18	CAGGGCTTGAGCTCTGAT	RP11-130F10.1	NOVEL	antisense	0	0	0	0	0
@SRR360717.53665517-A	chr4	179025886	179025906	21	GTGTGTGTGTGTGTGTATATA	0	0	0	0	0	0	0	0
@SRR023393.7942056	chr4	182127602	182127626	25	AAAATACAGATCCTTCCATAAAATA	0	0	0	0	0	0	0	0
@ERR044536.11787792-A	chr4	184813976	184813995	20	CTGTTACCTACTAATTGACA	STOX2	KNOWN	protein_coding	0	0	0	0	0
@ERR043037.42935654	chr4	187830018	187830040	23	TATGACATTAAAAGCATGTCATA	0	0	0	0	0	0	0	0
@ERR018508.11062802-B	chr4	188994088	188994112	25	AAACCTCCTTTAATGAATAGTTTTT	0	0	0	0	0	0	0	0
@SRR065438.11462102	chr5	10770725	10770743	19	GTCTCTGTGTGTGTGTGTG	0	0	0	0	0	0	0	0
@SRR065438.11462102	chr5	10770755	10770769	15	GTGTGTGTGTGTGTG	0	0	0	0	0	0	0	0
@SRR014776.7479955	chr5	12693290	12693314	25	TTTACAAATTACAAAAATTACAAAA	CT49	NOVEL	lincRNA	0	0	0	0	0
@ERR009211.1973325-B	chr5	15840449	15840482	34	TTGGAAAAGAATGAGGAGGAGGGGTTTTTACTAA	FBXL7	KNOWN	protein_coding	0	0	0	0	0
@SRR062598.13971566-B	chr5	24935858	24935879	22	TGTGTGTGTGTGTGTGTGTATA	0	0	0	0	0	0	0	0
@SRR360717.58481418-B	chr5	29832459	29832480	22	TATACACACACACACACACACA	0	0	0	0	0	0	0	0
@SRR029766.4905999	chr5	36168878	36168913	36	AGAGAAAATAGACATGTCTTGCTCAATGTTTCCTCT	SKP2	KNOWN	protein_coding	0	0	0	0	GATA3
@ERR005766.1035351-B	chr5	43265098	43265123	26	GTTACCCACAAAGGGAAGCCCATCAG	NIM1	KNOWN	protein_coding	0	0	0	0	0

@SRR350142.142747053-A	chr5	49771656	49771675	20	GCTTCACCTGAGATAAGACA	0	0	0	0	0	0	0	0
@ERR013130.14396108-A	chr5	50772789	50772807	19	AAAAAAAAAAAAAAAAAAA	0	0	0	0	0	0	0	0
@SRR063073.18303719-B	chr5	51110451	51110470	20	TATTACTATACTAGTAATAA	0	0	0	0	0	0	0	0
@SRR062572.7517540-A	chr5	51110472	51110491	20	TTATTACTAGTATAGTAATA	0	0	0	0	0	0	0	0
@SRR068160.105081486-A	chr5	64364621	64364639	19	TCTCTTGACTTAAGCAATG	0	0	0	0	0	0	0	0
@SRR023379.2961196	chr5	66320082	66320120	39	GAACAGAGTTCCATCGTGTGTGTGGATGGAATACTGTTC	MAST4	KNOWN	protein_coding	0	0	0	0	0
@SRR023389.14227164-B	chr5	67501817	67501835	19	CTTTTTTTTTTTTTTTTTT	0	0	0	0	0	0	0	0
@SRR022654.5152240-B	chr5	79952851	79952870	20	AGAATTTTCTATTAAGAAAA	MSH3	KNOWN	protein_coding	0	0	0	0	0
@SRR043208.18296301-A	chr5	80361097	80361129	33	AAGCAGAACTGAAGCCACTAAATTTGGGGATAA	RASGRF2	KNOWN	protein_coding	0	0	0	0	0
@ERR009407.8386778-A	chr5	87084867	87084883	17	AAAAAAAAAAAAAAAAA	0	0	0	0	0	0	0	0
@SRR017294.16808138-A	chr5	100581880	100581896	17	ACACACACACACACACT	0	0	0	0	0	0	0	0
@SRR015526.3738741-A	chr5	102093782	102093803	22	AAAAAGAAAAAAAAAAAAAAAA	PAM	KNOWN	protein_coding	0	0	0	0	0
@SRR360763.59062574-B	chr5	103305226	103305258	33	TGTATTAATCTCTCTTACTAGACTGTGAGATTA	0	0	0	0	0	0	0	0
@ERR043017.75211-A	chr5	113226028	113226053	26	GACCTCAAGTGATCCACCCACCTCAG	0	0	0	0	0	0	0	0
@ERR042533.102701991-A	chr5	114280354	114280377	24	TATATACACACACTGTATATACTG	0	0	0	0	0	0	0	0
@ERR042533.102701991-A	chr5	114280391	114280408	18	TATACACACACTGTATAT	0	0	0	0	0	0	0	0
@ERR042955.38465811-A	chr5	114280412	114280431	20	TATACACACACTGTATATAC	0	0	0	0	0	0	0	0
@SRR023366.8721268	chr5	115716024	115716050	27	TCTTCTGACCTAATGAAGGTCAGAAGA	COMMD10	KNOWN	protein_coding	0	0	0	0	0
@ERR006199.15988967-B	chr5	125545922	125545945	24	TTTGGCAGTTGATNCTCCTGCCCA	RP11-114J13.1	NOVEL	lincRNA	0	0	0	0	0
@SRR014615.6718325-A	chr5	128464661	128464680	20	AAAGCCAAGATCCTGGCTCT	0	0	0	0	0	0	0	0
@ERR016162.12702010-B	chr5	132544125	132544158	34	AGTTTAAAAGGAATCTACAAGTCCAGGCTAATCT	CTB-49A3.2	NOVEL	antisense	0	0	0	0	0
@ERR016162.12702010-B	chr5	132544125	132544158	34	AGTTTAAAAGGAATCTACAAGTCCAGGCTAATCT	FSTL4	KNOWN	protein_coding	0	0	0	0	0
@ERR018558.11437555-B	chr5	139253306	139253328	23	CACAGCAGTGGGCAGTCTGTGCC	NRG2	KNOWN	protein_coding	0	0	0	0	0
@ERR016160.1877020-B	chr5	140055481	140055496	16	GGGCGGAAACCACCCA	HARS	KNOWN	protein_coding	1	0	0	0	0
@SRR029674.21956139-A	chr5	144021197	144021213	17	CAAATATAAAAAGCAGT	0	0	0	0	0	0	0	0
@ERR013116.14326828	chr5	163470232	163470253	22	CAGGTTCAAAATTAGCAAACTG	0	0	0	0	0	0	0	0
@SRR029745.24230264-A	chr5	164636374	164636404	31	TAACAAAATAGTAAGTTTTTTGTATTTGCTA	0	0	0	0	0	0	0	0
@ERR009293.4755895-B	chr5	178433551	178433572	22	CTCTCTCTCTCTCTCTCTCTTT	0	0	0	0	0	0	0	0
@ERR009293.4755895-B	chr5	178433573	178433596	24	CTCTCTCTCTCTCTAGCTCTCTCT	0	0	0	0	0	0	0	0
@ERR009371.12844294-B	chr5	179256680	179256707	28	TAGGAGGAGAGGAAAGATAAAAAAACTA	SQSTM1	KNOWN	protein_coding	0	0	0	0	0

@SRR029678.7160280	chr5	179633644	179633663	20	TCTCCTTAAATTTAAAAAGA	RASGEF1C	KNOWN	protein_coding	0	0	0	0	0
@SRR359106.164775077	chr6	3027009	3027032	24	CATCAAACACATCACTGGATGTGT	RP1-90J20.11	NOVEL	sense_overlapping	1	0	0	multiple	0
@ERR019903.32557563-A	chr6	3711172	3711193	22	TTTATATTTCTAAGTGAGTGTA	0	0	0	0	0	0	0	0
@SRR014687.5812080-A	chr6	3715875	3715903	29	TTCTATGTATACTTCTCTATACCTTGCTA	0	0	0	0	0	0	0	ESR1
@ERR020257.20196206	chr6	4365708	4365734	27	TACCTTTCTTACTAACCTCTCACTGTA	0	0	0	0	0	0	0	0
@SRR031343.4228212	chr6	6726440	6726466	27	TAATGCTATTGGAAACTGGAAGCACTA	0	0	0	0	0	0	0	CTCF,EGR1,MYC,POLR2A
@SRR026656.16174048-A	chr6	11708263	11708286	24	AAACTTGCTTTATGTCTTTTATTT	0	0	0	0	0	0	0	0
@ERR022461.57924697-A	chr6	12884853	12884872	20	ATCCTGACGTCAGGAGATCG	PHACTR1	KNOWN	protein_coding	0	0	0	0	0
@ERR020276.74360741-B	chr6	15470587	15470607	21	TCTGCCTCAATCACTTACAAA	JARID2	KNOWN	protein_coding	0	0	0	0	0
@ERR012118.5729500	chr6	18111496	18111523	28	CTCTCTCTCTCGAGAGAGAGAGAGAGAG	0	0	0	0	0	0	0	0
@ERR012620.1724725	chr6	18125251	18125284	34	TCTAGGTGGAAGCAGGAAAAGATGGGCATGCTAG	0	0	0	0	0	0	0	0
@SRR015528.12951448	chr6	20825168	20825191	24	ATTAGGAAAAAGAAGCAAGATATA	CDKAL1	KNOWN	protein_coding	0	0	0	0	0
@ERR005703.10706868	chr6	23710810	23710838	29	AGATTGGTCCAAATTTAATTCCACAATCT	0	0	0	0	0	0	0	0
@ERR009365.11005372-A	chr6	24571645	24571668	24	GCAAACATTGGGTAATGGCTGCCA	KIAA0319	KNOWN	protein_coding	0	0	0	0	0
@SRR069525.98059749-A	chr6	25016116	25016135	20	AAAAAAAAAAAAAAAAAAAA	FAM65B	KNOWN	protein_coding	0	0	0	0	0
@SRR069525.98059749-A	chr6	25016116	25016135	20	AAAAAAAAAAAAAAAAAAAA	RP11-367G6.3	NOVEL	lincRNA	0	0	0	0	0
@ERR013101.5359840-A	chr6	26651238	26651268	31	ACTTCAAATAGTATAGTAAGTGTTGAAGTTA	ZNF322	KNOWN	protein_coding	0	0	0	0	0
@SRR360763.219260812-B	chr6	32504267	32504287	21	TTTTTAAAAAAAAAAAAAAAA	0	0	0	0	0	0	0	0
@SRR016211.148112	chr6	39867720	39867751	32	CTTTGGAAGAGGGAACACCCTGCATCTCAAAG	DAAM2	KNOWN	protein_coding	0	0	0	0	0
@SRR016211.148112	chr6	39867720	39867751	32	CTTTGGAAGAGGGAACACCCTGCATCTCAAAG	RP11-61I13.3	NOVEL	antisense	1	0	0	0	0
@SRR016211.148112	chr6	39867720	39867751	32	CTTTGGAAGAGGGAACACCCTGCATCTCAAAG	MOCS1	KNOWN	protein_coding	0	0	0	0	0
@SRR032328.2807243-A	chr6	39902756	39902788	33	CTTGCCTTCTCCACGAAAAAAAAAAAAAAAAAA	0	0	0	0	0	0	0	0
@ERR018545.14098611-B	chr6	39950035	39950062	28	GCCAATCAGAGCAACAGTAATGATTGGC	0	0	0	0	0	0	0	0
@SRR032223.5528218-B	chr6	39980482	39980503	22	TGTACATATTTGGGGAGTACAT	0	0	0	0	0	0	0	0
@ERR005767.14237262-A	chr6	42333837	42333875	39	CCACCCAGCAGACTGCAGGAGAGGGCTGGAGCTGGGTGG	TRERF1	KNOWN	protein_coding	0	0	0	0	0
@SRR006186.3876141	chr6	47347183	47347220	38	ACTTGTGTATGTATGCAGATTTAACATTCAAACCAAGT	0	0	0	0	0	0	0	CEBPB,F

													OS
@ERR009218.2102103-B	chr6	54225639	54225654	16	AAAAAAAAAAAAAAAA	TINAG	KNOWN	protein_coding	0	0	0	0	0
@SRR029764.17358775	chr6	57363454	57363479	26	ATGACAATTCAAGAGGAATTGTCATG	PRIM2	KNOWN	protein_coding	0	0	0	0	0
@ERR020234.83067995-B	chr6	63560660	63560681	22	CTGTATCACAGGGGGTGTACAC	0	0	0	0	0	0	0	0
@SRR360755.8785685-B	chr6	64781455	64781487	33	CTTTAAGATTTAGCTGATTTTTCTTTGTTAAAG	EYS	KNOWN	protein_coding	0	0	0	0	0
@ERR018538.3925514-B	chr6	70493915	70493948	34	AGCCAAATACACTAACAGTACTTGAGTGTATTTG	LMBRD1	KNOWN	protein_coding	0	0	0	0	0
@SRR360641.32073976-B	chr6	72429360	72429389	30	TGCAGTGAGCTGAGATCATGCCACTGCACT	0	0	0	0	0	0	0	0
@SRR064192.80674470	chr6	75780718	75780738	21	TAAATTTAAGAATCCTAAAAT	0	0	0	0	0	0	0	0
@SRR029933.11831294	chr6	81479826	81479860	35	AACTTTACATTCCTAGAGAAAGAAGATTTTATTTT	0	0	0	0	0	0	0	0
@SRR014723.5256509	chr6	94013905	94013926	22	CTTGCTCAATGTATACTACAAG	EPHA7	KNOWN	protein_coding	0	0	0	0	0
@ERR042944.46277738	chr6	113200735	113200759	25	ATAATACCCTTCAACAATATCTTAA	0	0	0	0	0	0	0	0
@ERR018499.2705866-A	chr6	113201661	113201684	24	CTGCTGCCATTACCCCGGCTGCAG	0	0	0	0	0	0	0	0
@SRR014696.12141409	chr6	117221746	117221770	25	AAAAATTTTTTTAAATAAAAATAG	RFX6	KNOWN	protein_coding	0	0	0	0	0
@SRR062641.272273-A	chr6	118618828	118618850	23	TATATTTCTCAGAACTTATATAA	SLC35F1	KNOWN	protein_coding	0	0	0	0	0
@SRR006215.12711609	chr6	125139120	125139144	25	TAAATAATGACCACTCAAGTGCATG	NKAIN2	KNOWN	protein_coding	0	0	0	0	0
@SRR014649.3393831-A	chr6	130387969	130387993	25	CCAGTTAACACTAAACTTATTTATA	L3MBTL3	KNOWN	protein_coding	0	0	0	0	0
@SRR014703.6941736-B	chr6	141004965	141005002	38	AGACTACTCCAGGTTACATAGTAAACCTCAAATAGCGT	MIR4465	KNOWN	miRNA	1	0	0	0	0
@SRR015989.8787501	chr6	144712129	144712147	19	ACAACTAATTGTTATTAAA	UTRN	KNOWN	protein_coding	0	0	0	0	0
@ERR018540.7479539-A	chr6	150565380	150565407	28	TAATGGATCGGGCAAGAAAGGGCCAGTG	PPP1R14C	KNOWN	protein_coding	0	0	0	0	ZNF263
@ERR013116.17070247	chr6	150577686	150577709	24	TCATGGGTCAAATGACTCTCATGA	0	0	0	0	0	0	0	0
@ERR016137.17478659-A	chr6	150692316	150692334	19	ACATTAAGAGCGAGTAATA	IYD	KNOWN	protein_coding	0	0	0	0	0
@SRR029713.3220093-A	chr6	151077056	151077092	37	TATCTTGTAGTAAATGCAGCTTCTATCTACCACAAGA	PLEKHG1	KNOWN	protein_coding	0	0	0	0	0
@ERR009178.5224344-B	chr6	154021719	154021752	34	AGACTGCTTTCCCAGAGAACAGTGAAATATTACA	0	0	0	0	0	0	0	0
@SRR029728.11009857	chr6	159045354	159045386	33	ACTTGGGATCATTTTTCTCAAAAGCACCTGATT	TMEM181	KNOWN	protein_coding	0	0	0	0	0
@ERR018492.12222009-B	chr6	165028234	165028253	20	TCCTTCTAAAGATTAAGAAA	0	0	0	0	0	0	0	0
@SRR360543.30142115-A	chr7	108375	108403	29	GCTTGGGTTGAGTGGGCAGATGGACGTGC	0	0	0	0	0	0	0	CTCF,MAX
@ERR016343.5522469-B	chr7	1215535	1215566	32	CCGCTGAGCTGGAAAGGGCCTTTTCCCATCCC	0	0	0	0	0	0	0	GATA2,PHF8,

													POLR2A,SIN3A
@ERR013106.1524898-B	chr7	3079914	3079932	19	GCACTATCTGCTTTGCAGG	CARD11	KNOWN	protein_coding	0	0	0	0	0
@SRR029828.19433537	chr7	3155640	3155677	38	TGACTTAGTTGAACCAAGAGGGAAGTTGAACTAAGCCA	0	0	0	0	0	0	0	0
@ERR018540.10411687-A	chr7	3467785	3467807	23	GTCACTTCTTTCAAAGCCTGTCA	SDK1	KNOWN	protein_coding	0	0	0	0	SP1
@SRR111942.43445196-A	chr7	3572737	3572764	28	CTCCACCTGAATCACATGGTTTAGGTTG	SDK1	KNOWN	protein_coding	0	0	0	0	0
@SRR111942.64811216-A	chr7	4309428	4309454	27	AAGATGGGTGATAAGGGGGCAGCCATG	0	0	0	0	0	0	0	0
@SRR350153.31122242-A	chr7	8868223	8868241	19	TATAACACAAAATTTTGAC	0	0	0	0	0	0	0	0
@ERR016235.9428012-A	chr7	8965088	8965111	24	TATATACACACAAACAACATATAA	0	0	0	0	0	0	0	0
@SRR061660.6487361-A	chr7	9090814	9090849	36	ATGTTAGAATTACCTGAGGAAGATTTTAAAACACAT	0	0	0	0	0	0	0	0
@SRR029727.4584804	chr7	9995539	9995577	39	GATAATTTTCTTTGTCTTCCCCACTCAAAGCTGTTTAAC	0	0	0	0	0	0	0	0
@SRR063281.4059663-B	chr7	15411533	15411557	25	TATTTAGAAACACTTAATAATTTAA	AGMO	KNOWN	protein_coding	0	0	0	0	0
@SRR063412.2063975	chr7	27614111	27614137	27	TAAAAAAGGTAAAGAGTTACCTGTTTG	HIBADH	KNOWN	protein_coding	0	0	0	0	0
@SRR016211.3552776-A	chr7	28956215	28956239	25	AAAAAAAAAAAAAAAAAAAAAAAAA	0	0	0	0	0	0	0	0
@SRR023858.14667927-B	chr7	29166632	29166668	37	AGTGATGACTAATTCGTTAGAGAGATTAGACATCACT	CPVL	KNOWN	protein_coding	0	0	0	FOS	0
@SRR023858.14667927-B	chr7	29166632	29166668	37	AGTGATGACTAATTCGTTAGAGAGATTAGACATCACT	CHN2	KNOWN	protein_coding	0	0	0	FOS	0
@SRR359110.135053576-A	chr7	31426377	31426398	22	TATACACACATACACACACATA	0	0	0	0	0	0	0	0
@SRR062545.12783686-A	chr7	31658252	31658274	23	TAATTATGGGTTTCTACATTGAG	CCDC129	KNOWN	protein_coding	0	0	0	0	0
@ERR020261.89922271-A	chr7	34381068	34381087	20	AGGATGTAAATTACCTTCCT	0	0	0	0	0	0	0	EP300,FOS,FOXA1,GATA3,TCF7L2
@ERR018479.19850616-B	chr7	45388205	45388239	35	AACACCTGGAACCACTCTGGCACACAACAGGTGTT	0	0	0	0	0	0	0	0
@SRR063407.26938308	chr7	53646579	53646599	21	GTGTATGTGTGTGTGTATATA	0	0	0	0	0	0	0	0
@SRR043396.12725599-A	chr7	54111907	54111922	16	GATCAACAAAATTGAT	0	0	0	0	0	0	0	0
@SRR061640.4073605-B	chr7	70304578	70304597	20	TTCATTGCTTTATTTCAAAG	0	0	0	0	0	0	0	0
@SRR016204.1879781	chr7	70772689	70772723	35	TCCTTCTAATGAGTATGCTTAACTTGGTAGAAGGA	WBSCR17	KNOWN	protein_coding	0	0	0	0	0
@SRR016204.1879781	chr7	70772689	70772723	35	TCCTTCTAATGAGTATGCTTAACTTGGTAGAAGGA	MIR3914-1	KNOWN	miRNA	1	0	0	0	0

@ERR018442.33841382-A	chr7	70878844	70878870	27	ATACACTGTTCCAAACAACA GTGTGGG	WBSCR17	KNOWN	protein_coding	0	0	0	0	0
@SRR029886.8331020	chr7	96609331	96609349	19	ATCTCAGAGTTAGAAGACT	DLX6-AS1	KNOWN	antisense	0	0	0	0	0
@SRR014706.8760603-B	chr7	96791898	96791928	31	TGTTCTTTGAAACCAATGAGA ACAAAGCCAC	ACN9	KNOWN	protein_coding	0	0	0	0	0
@ERR018450.37627657-A	chr7	96825024	96825048	25	GAGCTTCTGCACAACAAAAG AAACT	0	0	0	0	0	0	0	0
@SRR027544.884418-A	chr7	101012166	101012191	26	AGCCAGATTTTGAGTGAGTTT TTTCT	COL26A1	KNOWN	polymorphic_pseudogene	0	0	0	0	0
@SRR014199.5291791	chr7	101154153	101154177	25	AAAAAGAGAAAAAGAAAAA AATTGG	COL26A1	KNOWN	polymorphic_pseudogene	0	0	0	0	0
@ERR043038.37640771	chr7	107224415	107224457	43	TATTTATTCAAAGAAGGGAT CTAGTACTTACCTAGAAATA GAA	BCAP29	KNOWN	protein_coding	1	1	1	0	0
@ERR012621.8594551-B	chr7	107410668	107410682	15	AAAAAAAAAAAAAAA	SLC26A3	KNOWN	protein_coding	0	0	0	0	0
@SRR065443.5001659	chr7	111363320	111363353	34	TACTTTTTTATTTAGAGATAT AAGGTAAACATTA	0	0	0	0	0	0	0	0
@SRR014132.1893648	chr7	112963904	112963923	20	TTTTATTTTTTATTTTTATT	0	0	0	0	0	0	0	0
@ERR018527.11343239-A	chr7	115985936	115985955	20	TATATACACACACACACACA	AC002066.1	NOVEL	antisense	0	0	0	0	0
@ERR018527.11343239-A	chr7	115985936	115985955	20	TATATACACACACACACACA	CAV2	KNOWN	protein_coding	0	0	0	0	0
@ERR018527.11343239-A	chr7	115985936	115985955	20	TATATACACACACACACACA	AC002066.2	NOVEL	miRNA	1	0	0	0	0
@SRR350153.42353736-B	chr7	117145509	117145527	19	GCAAATTCCACGAGGTGGC	CFTR	KNOWN	protein_coding	0	0	0	0	CTCF,RAD21,SMC3
@ERR013091.6205414-A	chr7	117357033	117357067	35	TTGTAAGCCACCTTGGGAGT AGATGAGAACGGCAA	CTTNBP2	KNOWN	protein_coding	0	0	0	0	0
@ERR042512.53109194-A	chr7	118367021	118367035	15	GTAGTTAAATACTAC	0	0	0	0	0	0	0	0
@SRR101465.10691120-A	chr7	120481956	120481983	28	TTAGAAGAAAGCTTCCCTGG AAACCTTC	TSPAN12	KNOWN	protein_coding	0	0	0	0	0
@ERR018202.31110005	chr7	136559881	136559899	19	TGTGTGTGTGTGTGTGTGT	hsa-mir-490	NOVEL	antisense	0	0	0	0	0
@ERR018202.31110005	chr7	136559881	136559899	19	TGTGTGTGTGTGTGTGTGT	CHRM2	KNOWN	protein_coding	0	0	0	0	0
@SRR014705.3662599-A	chr7	152364506	152364524	19	TTATTTTTTTTTTCTTTTT	XRCC2	KNOWN	protein_coding	0	0	0	0	CEBPB
@ERR012160.21081953	chr8	3702413	3702446	34	TGGCAGTTTTTAAATGCACAG ACAAATATTGACA	CSMD1	KNOWN	protein_coding	0	0	0	0	0
@SRR029763.17485582	chr8	4318169	4318206	38	TGTTTATAAAATTCTCTTACA GTCTTCTTCTTAAACAT	CSMD1	KNOWN	protein_coding	0	0	0	0	0
@SRR063093.15752119-B	chr8	11703579	11703608	30	TGAATCAGTCATCTAGCATTA AATGTTTTA	CTSB	KNOWN	protein_coding	0	0	0	0	0
@SRR029832.9757886	chr8	17866907	17866933	27	TTAGTATTACTAAAGAATACT GCTATA	PCM1	KNOWN	protein_coding	0	0	0	0	0
@SRR015498.1883889-A	chr8	19406058	19406088	31	AATGTTTTAAAAATAATATTT GAGAAAAACT	CSGALNACT1	KNOWN	protein_coding	0	0	0	0	0
@SRR029911.12339390-B	chr8	21141585	21141609	25	CACTTGAGCCCAAGAGGCTC AAGTG	0	0	0	0	0	0	0	0
@SRR027523.6437954-B	chr8	27186243	27186274	32	TGTGCATGGGCTCTACAGCC ATCCNCAGGGAA	PTK2B	KNOWN	protein_coding	0	0	0	MAX,MYC	0

@ERR013128.25806890-A	chr8	30531151	30531165	15	GTGTGTGTGTGTACA	0	0	0	0	0	0	0	0
@ERR013128.25806890-A	chr8	30531177	30531194	18	TGTGTGTATGTGTTTGTG	0	0	0	0	0	0	0	0
@SRR031344.22244713	chr8	33648918	33648947	30	ATATACACACACACACACAC ACACACACAT	RP11-317N12 .1	NOVEL	lincRNA	0	0	0	0	0
@SRR360753.49639573-A	chr8	40678372	40678390	19	ATATACACACACACACCCA	ZMAT4	KNOWN	protein_coding	0	0	0	0	0
@SRR062664.21530677	chr8	42578141	42578160	20	TTTTAGTAAATATCTAAGCA	CHRNB3	KNOWN	protein_coding	0	0	0	0	0
@ERR022463.60546708-B	chr8	43092950	43092969	20	TAAAATATCAAAGTACCCAA	0	0	0	0	0	0	0	0
@ERR018545.32532733-B	chr8	43093132	43093151	20	TAATATACTGTACACAAAAT	0	0	0	0	0	0	0	0
@SRR062595.9093555-A	chr8	43093962	43093978	17	TGTATTTGGTGTACTTT	0	0	0	0	0	0	0	0
@ERR018539.16026357-B	chr8	43094974	43094996	23	TACTTTGGGTACTTTGATATT TT	0	0	0	0	0	0	0	0
@ERR015505.9491073-B	chr8	43095094	43095111	18	GGGTACTTTGATATTGTA	0	0	0	0	0	0	0	0
@SRR111943.93544078-B	chr8	43095180	43095200	21	ACTTTGGGTACTTTGATATTT	0	0	0	0	0	0	0	0
@ERR022462.63280140-B	chr8	43096615	43096638	24	TACTTTGGGTACTTTGATATT TTA	0	0	0	0	0	0	0	0
@ERR020283.12358274-B	chr8	43096657	43096680	24	TACTTTGGGTACTTTGATATT TTA	0	0	0	0	0	0	0	0
@ERR018539.5501479	chr8	52230395	52230420	26	GTCCCAGCTACTCGGGAGGC TGAGGC	RP11-401H2. 1	NOVEL	antisense	0	0	0	0	0
@SRR029916.13959877	chr8	52485223	52485243	21	AACATTAAGTACTAAGCAGT A	PXDNL	KNOWN	protein_coding	0	0	0	0	0
@SRR111943.77645173-A	chr8	53973058	53973081	24	ACTTAACTTAGAATAAGATT AAGT	0	0	0	0	0	0	0	0
@SRR061683.8819639-A	chr8	67280276	67280299	24	ACAACATTTTCCATTAGGAA AATG	0	0	0	0	0	0	0	0
@ERR009273.10555236-A	chr8	74575787	74575811	25	CTTCTGCAAATTGTTTTTGCA GTAG	STAU2	KNOWN	protein_coding	0	0	0	0	0
@ERR018555.2046411-A	chr8	83364322	83364347	26	CCTGTGCCCAGTGCTGATGGC ACAGG	0	0	0	0	0	0	0	0
@ERR018492.58034467-B	chr8	86283638	86283657	20	GTGTGTATACCCACATACTC	CA1	KNOWN	protein_coding	0	0	0	0	0
@SRR015987.5233085	chr8	95627808	95627828	21	TTGAAATTTTTTTTTTTTTAA	0	0	0	0	0	0	0	0
@ERR018542.4138159-A	chr8	108555611	108555632	22	TTTGCTGTGAAATAAATTTAC A	0	0	0	0	0	0	0	0
@SRR017510.7946343	chr8	122563231	122563257	27	AGTAATTGAGATTTTTGCCAA ATTTCT	0	0	0	0	0	0	0	0
@SRR063405.3717921	chr8	123238524	123238556	33	TTGCCCATAACCCTTGGGGCT ATAAATACCCAA	0	0	0	0	0	0	0	0
@SRR038701.4367365-B	chr8	128181988	128182017	30	TAATAGAACTACCTAATAGT AGTTCTATTA	0	0	0	0	0	0	0	0
@ERR018438.13522537-A	chr8	132936264	132936283	20	GTATCAGGGACTTACTGATA	EFR3A	KNOWN	protein_coding	0	0	0	0	0
@SRR017031.4054346	chr8	133370910	133370930	21	CACCAAGTGCTGGGGACATT C	KCNQ3	KNOWN	protein_coding	0	0	0	0	0
@SRR065200.11691966-B	chr8	134347448	134347474	27	TGTGTGTTTACACGATGAAA GCACACA	0	0	0	0	0	0	0	0
@SRR019043.1954641	chr8	135446932	135446950	19	TCACCCCTTAAGGAGCACA	0	0	0	0	0	0	0	0
@ERR015743.5010410	chr8	136540151	136540181	31	TTTTAATAAAGGATACACTAC GTTATTAAAA	KHDRBS3	KNOWN	protein_coding	0	0	0	0	0

@SRR360610.136603186-A	chr8	140122514	140122536	23	TACAGTACGCATTGGAAATGTAA	0	0	0	0	0	0	0	0
@SRR032295.7355794-A	chr8	140184187	140184217	31	ATGTTTACCGGAAGCATTAGAGTTATTGTAT	0	0	0	0	0	0	0	0
@ERR038224.18923940	chr8	140511804	140511828	25	TGCTGATCCATCCACAGGAATGCTC	0	0	0	0	0	0	0	0
@SRR359083.47301732-A	chr8	140531116	140531140	25	TATTTTTTTTTTTTTTTTTTTTTTT	0	0	0	0	0	0	0	0
@SRR015499.2019591-A	chr8	140574358	140574388	31	AGTTTATCTCAGAAAAAAAAAAGCAAAAATT	0	0	0	0	0	0	0	0
@SRR063411.22282843	chr8	142478529	142478546	18	CCCTGGCCACAGGGAGGC	MROH5	KNOWN	polymorphic_pseudogene	0	0	0	0	0
@ERR009331.7124423-A	chr9	2570226	2570244	19	CACATGTGTACATACACAT	RP11-125B21.2	NOVEL	antisense	0	0	0	0	0
@SRR006274.7155664	chr9	5784744	5784769	26	TGTTTGTTTTTACAGTTTAGCATATA	ERMP1	KNOWN	protein_coding	1	0	1	0	0
@ERR016092.18787083	chr9	7742380	7742399	20	GGGTTTTTTTTTTTTTTTTT	0	0	0	0	0	0	0	0
@SRR061669.27821601-A	chr9	13766936	13766972	37	TAGTTTCCTTTTTTTGAAATCAAAGAATTAGGTAATA	0	0	0	0	0	0	0	0
@SRR014130.9751418	chr9	15864580	15864611	32	TCGGCACAATTGGAGAAGCTCTAATTAAACTA	CCDC171	KNOWN	protein_coding	0	0	0	0	0
@SRR233125.189310214-A	chr9	25554050	25554067	18	ATAGAGTTTGATCATTTT	0	0	0	0	0	0	0	0
@SRR017038.15588016	chr9	28014540	28014568	29	TTGTCTTATGCTGGATAAAAAAGTAACAT	LINGO2	KNOWN	protein_coding	0	0	0	0	0
@SRR360757.186110885-A	chr9	31249888	31249908	21	TTGGTCTTTATTTTCAATTGA	0	0	0	0	0	0	0	0
@SRR014694.6335282-A	chr9	34867425	34867444	20	GTAGACCCTGAAGGGGTCTA	0	0	0	0	0	0	0	0
@SRR043408.13179321-A	chr9	73039279	73039296	18	AAAAAAAAAAAAAAAAAG	0	0	0	0	0	0	0	0
@SRR029773.1020048	chr9	73057279	73057297	19	TTCTATTTTTAAATAAAAA	0	0	0	0	0	0	0	0
@SRR029855.9464105-A	chr9	73792532	73792557	26	AATCTTAGGTTTTCCTAATACATGAA	TRPM3	KNOWN	protein_coding	0	0	0	0	0
@SRR360715.129495168-A	chr9	83608718	83608739	22	TATATACACACCCTGTTGTGTA	0	0	0	0	0	0	0	0
@SRR359096.144921498-A	chr9	83608740	83608766	27	ATATACACAACAGGGTGTGTGTATATA	0	0	0	0	0	0	0	0
@SRR023342.5797784-A	chr9	83608781	83608801	21	CACACACACAACAGGGTGTGT	0	0	0	0	0	0	0	0
@ERR013040.11737604-A	chr9	83608812	83608829	18	CAACAGGGTGTGTATATA	0	0	0	0	0	0	0	0
@ERR019494.16137115-B	chr9	83608854	83608871	18	TTTGTGTGTGTGTGTATA	0	0	0	0	0	0	0	0
@SRR062628.5297146-A	chr9	92084241	92084281	41	TAGTTTTACTGGAAAATTCTACCAAACATGTAAGGAACCTA	SEMA4D	KNOWN	protein_coding	0	0	0	0	0
@SRR062627.11964412-B	chr9	111554646	111554673	28	GGTAAAAAGACATTATTCAGAAAGAACC	0	0	0	0	0	0	0	0
@SRR359939.27408126-A	chr9	112164549	112164577	29	ATAAAAAAAAAAAGGAAATGTAGAGGTTAT	PTPN3	KNOWN	protein_coding	0	0	0	0	0
@SRR061675.12337745-A	chr9	113134132	113134151	20	TAGAGAGATTCATTGCGGGA	SVEP1	KNOWN	protein_coding	0	0	0	0	multiple
@ERR022462.63476659-A	chr9	116977166	116977196	31	GCTCAGTCTCTCACTGTGAGTGATACTGAGC	COL27A1	KNOWN	protein_coding	0	0	0	0	0
@SRR360588.138181284-A	chr9	118567268	118567289	22	TATATACGTGTGTGTATATACA	0	0	0	0	0	0	0	0

@SRR031624.20181691-B	chr9	128316796	128316829	34	GTTAGGATTACAGGCATGAGCCACCGCTCCTAAC	MAPKAP1	KNOWN	protein_coding	0	0	0	0	0
@ERR022463.60368983-A	chr9	129855907	129855929	23	GCCCACTCAGAGGTACATGCTGA	RALGPS1	KNOWN	protein_coding	0	0	0	0	0
@ERR022463.60368983-A	chr9	129855907	129855929	23	GCCCACTCAGAGGTACATGCTGA	ANGPTL2	KNOWN	protein_coding	0	0	0	0	0
@ERR013096.19971098-B	chrM	1336	1368	33	TGTAGCCCATTTCTTGCCACCTCATGGGCTACA	MT-RNR1	KNOWN	Mt_rRNA	1	0	0	0	0
@ERR052834.42864962-B	chrX	9307553	9307572	20	TTTTTTTTTTTTTTTTTTTT	0	0	0	0	0	0	0	0
@ERR018499.19098057-A	chrX	10105150	10105170	21	CACACACACACACACACACAC	WWC3	KNOWN	protein_coding	0	0	0	0	0
@ERR013123.12419950-A	chrX	14218576	14218597	22	ATGTGTCTTTATAGCAGCATGA	0	0	0	0	0	0	0	0
@SRR029778.13231557-A	chrX	16176518	16176546	29	AGATACCTAAGTCCATATCTGAGTTTCTT	RP11-431J24.2	NOVEL	antisense	0	0	0	0	0
@SRR032323.3054905-B	chrX	16869463	16869484	22	ACCAAATAATAACTTTTTTCTT	RBBP7	KNOWN	protein_coding	0	0	0	0	0
@ERR018479.30140539-B	chrX	25910278	25910298	21	CACGCAAAACAATGTTGTTGC	RP11-86A5.1	NOVEL	lincRNA	0	0	0	0	0
@ERR009320.4984168-A	chrX	27827629	27827647	19	GGGGGGGAGGGGCGGGGGG	MAGEB10	KNOWN	protein_coding	0	0	0	CTCF,GABPB1,POLR2A,REST	0
@ERR042508.40571350-B	chrX	27999208	27999232	25	AAGTTCAAGTGAAGACGTCGAACTT	DCAF8L1	KNOWN	protein_coding	1	1	0	0	0
@SRR031308.1350291	chrX	32756787	32756818	32	CTGCAGACGGGTGACAAGTGAACATGTAGAAG	DMD	KNOWN	protein_coding	0	0	0	0	0
@ERR012620.9954893-A	chrX	33027071	33027090	20	TATATACACACACGTATATA	DMD	KNOWN	protein_coding	0	0	0	0	0
@SRR350142.182768590-A	chrX	33028049	33028074	26	TATACACACACATGTGTGTATACACA	DMD	KNOWN	protein_coding	0	0	0	0	0
@SRR062594.7212726-A	chrX	40892680	40892697	18	TTTGTAACTTCACTTCAG	0	0	0	0	0	0	0	NFYA,NFYB,SP1
@ERR018465.9188350	chrX	40918483	40918502	20	TAACAAGTGATAATTTGTTA	0	0	0	0	0	0	0	0
@ERR020230.44563748-A	chrX	44514652	44514670	19	GAGGTCATGTACTCAAAAG	0	0	0	0	0	0	0	0
@ERR018558.18896877-B	chrX	75243503	75243533	31	TCATGAGTAGACTAGTGAATACACATGAGGG	0	0	0	0	0	0	0	0
@ERR020274.88992928-A	chrX	77853847	77853864	18	TTCGCACACAAAATTCAA	0	0	0	0	0	0	0	0
@ERR015880.21992085-B	chrX	78679652	78679671	20	TGAGGTGACATACATCCTCA	0	0	0	0	0	0	0	0
@SRR014842.7125911	chrX	93468113	93468141	29	GGTTTTTGCCACTTTTAATGGCAAAATCC	0	0	0	0	0	0	0	0
@SRR061646.12412325-B	chrX	95225111	95225136	26	TTAATTAGTATAGCTTATACCAATTA	0	0	0	0	0	0	0	0
@SRR022695.6052451-B	chrX	96432694	96432711	18	TTTTTAGTAGGTTTTTCA	DIAPH2	KNOWN	protein_coding	0	0	0	0	0
@ERR044613.52232623-B	chrX	101385429	101385455	27	ATCATGGGTCTGAGGAATTTGGGATGA	0	0	0	0	0	0	0	0
@ERR018483.69344360-B	chrX	106212095	106212116	22	TCTCCCTCTATTATTGTGTTAG	MORC4	KNOWN	protein_coding	0	0	0	0	0
@SRR032748.896223	chrX	106941773	106941806	34	GTTTTTTTTCCCATTAAAAGT	0	0	0	0	0	0	0	0

					AATGGTCAAAACC								
@ERR006197.15175265-A	chrX	121709217	121709244	28	ATAGCAAAACATTGAATAGG TGGCCTGT	0	0	0	0	0	0	0	0
@ERR009328.17692660-A	chrX	128071048	128071067	20	TGTGTGTGTATATACACACA	0	0	0	0	0	0	0	0
@SRR068147.39580105-A	chrX	132774606	132774627	22	CTTTAGCAATCCTAAAGTAA AA	GPC3	KNOWN	protein_coding	0	0	0	0	0
@ERR015505.2090603-A	chrX	136113611	136113635	25	CAACCGTTTTATCTTTAACCT CCTC	GPR101	KNOWN	protein_coding	1	1	0	0	0
@SRR360799.196927005-B	chrX	136196764	136196779	16	ATACACACACACACAC	0	0	0	0	0	0	0	0
@SRR359096.160043869-A	chrX	147662854	147662872	19	TATACACGTATACGTGTAC	AFF2	KNOWN	protein_coding	0	0	0	0	0
@ERR012115.12857132-B	chrX	153379714	153379736	23	ACAACAACGTAGATGGAGAA CCG	0	0	0	0	0	0	0	0
@SRR032322.7405077-A	chrY	59016094	59016122	29	TGTAATAAATGCTTGAGATTA GCAAACTG	0	0	0	0	0	0	0	0

Table S2. MIs and annotations detected from WXS LUSC CCLE data.

Name	Chr	Start	End	Length	MI	gene_name	gene_status	gene_type	exon	CDS	UTR	proximal_tfbs	distal_tfbs
@D0MUKACXX120302:8:2308:12828:188413-B	chr1	109824232	109824252	21	CAACATGAAGAGGGTGAGTTG	PSRC1	KNOWN	protein_coding	1	1	0	0	0
@C0FPWACXX120301:2:2307:6196:23792-A	chr1	186114826	186114857	32	GATAAGAAATTGGCTTCAAAATCTTCATAAAC	HMCN1	KNOWN	protein_coding	0	0	0	0	0
@D0MUKACXX120302:7:1104:5427:52231-B	chr1	227922788	227922817	30	CAGCCCTGCGTGTTTTCCAGCGCCTTCACG	SNAP47	KNOWN	protein_coding	1	0	1	multiple	0
@D0MUKACXX120302:7:1104:5427:52231-B	chr1	227922788	227922817	30	CAGCCCTGCGTGTTTTCCAGCGCCTTCACG	JMJD4	KNOWN	protein_coding	1	1	0	multiple	0
@C17JTACXX121021:1:2212:5801:69530-A	chr10	3176606	3176642	37	GTCATACAGTAACACTTTGCTGTTAAATAGAAATAAC	PFKP	KNOWN	protein_coding	0	0	0	0	0
@D0N5YACXX120305:4:2206:20871:133941-B	chr14	64988765	64988781	17	TTAGGAAAATTACCTGA	ZBTB1	KNOWN	protein_coding	1	1	0	0	0
@D0N5YACXX120305:4:2206:20871:133941-B	chr14	64988765	64988781	17	TTAGGAAAATTACCTGA	RP11-973N13.4	NOVEL	antisense	0	0	0	0	0
@D0N3RACXX120302:6:1306:4670:118510-B	chr15	40763870	40763903	34	CCGCTTCCAGTTAGAGCAGGCCACCTTGGGGACG	CHST14	KNOWN	protein_coding	1	1	0	multiple	0
@D0N3RACXX120302:7:1103:11306:61313-A	chr15	48063444	48063465	22	CATCTGCATGTCTCCCATGCTG	SEMA6D	KNOWN	protein_coding	1	1	1	0	0
@C0FJ4ACXX120306:1:1308:12036:27082-A	chr19	56389841	56389875	35	TATTATCCAAAAAAAGGTATGAAGCCGTCTCAGAT	NLRP4	KNOWN	protein_coding	0	0	0	0	0
@C17JTACXX121021:2:1313:10984:54747-A	chr2	64117420	64117448	29	TAATTACACTAGTAATTAAGGTGGCACTA	UGP2	KNOWN	protein_coding	0	0	0	0	0
@C0FJ4ACXX120306:2:1203:12810:153162-A	chr2	233391335	233391363	29	GTGTGAGGGCCAGGGCAACGTCCACACAC	CHRND	KNOWN	protein_coding	1	1	1	0	0
@C0J77ACXX120423:7:1212:13753:30384-B	chr20	62715296	62715326	31	GTCGGTGGAACCGGGGTACCGCATGCCCGAC	OPRL1	KNOWN	protein_coding	0	0	0	multiple	0
@C0J77ACXX120423:7:1212:13753:30384-B	chr20	62715296	62715326	31	GTCGGTGGAACCGGGGTACCGCATGCCCGAC	C20orf201	KNOWN	protein_coding	1	1	0	multiple	0
@C0FJ4ACXX120306:6:2304:17354:130068-A	chr5	131398448	131398468	21	GCTCAAAGTCGTCTGTTGAGC	IL3	KNOWN	protein_coding	1	1	0	0	0
